# A high throughput optical method for studying compositional effects in electrocatalysts for CO_2_ reduction

**DOI:** 10.1038/s41467-021-21342-w

**Published:** 2021-02-18

**Authors:** Jeremy L. Hitt, Yuguang C. Li, Songsheng Tao, Zhifei Yan, Yue Gao, Simon J. L. Billinge, Thomas E. Mallouk

**Affiliations:** 1 Department of Chemistry, The University of Pennsylvania, Philadelphia, PA USA; 2 Department of Chemistry, University at Buffalo, The State University of New York, Buffalo, NY USA; 3 Department of Applied Physics and Applied Mathematics, Columbia University, New York, NY USA; 4 Department of Mechanical Engineering, The Pennsylvania State University, University Park, State College, PA USA; 5 Condensed Matter Physics and Materials Science Department, Brookhaven National Laboratory, Upton, NY USA

**Keywords:** Electrocatalysis, Energy, Electrocatalysis, Nanoparticles

## Abstract

In the problem of electrochemical CO_2_ reduction, the discovery of earth-abundant, efficient, and selective catalysts is essential to enabling technology that can contribute to a carbon-neutral energy cycle. In this study, we adapt an optical high throughput screening method to study multi-metallic catalysts for CO_2_ electroreduction. We demonstrate the utility of the method by constructing catalytic activity maps of different alloyed elements and use X-ray scattering analysis by the atomic pair distribution function (PDF) method to gain insight into the structures of the most active compositions. Among combinations of four elements (Au, Ag, Cu, Zn), Au_6_Ag_2_Cu_2_ and Au_4_Zn_3_Cu_3_ were identified as the most active compositions in their respective ternaries. These ternary electrocatalysts were more active than any binary combination, and a ca. 5-fold increase in current density at potentials of −0.4 to −0.8 V vs. RHE was obtained for the best ternary catalysts relative to Au prepared by the same method. Tafel plots of electrochemical data for CO_2_ reduction and hydrogen evolution indicate that the ternary catalysts, despite their higher surface area, are poorer catalysts for the hydrogen evolution reaction than pure Au. This results in high Faradaic efficiency for CO_2_ reduction to CO.

## Abstract (web summary)

A high-throughput method is presented for the synthesis and testing of alloy electrocatalysts for gas phase CO_2_ electrolysis. Active ternary alloy catalysts were discovered and their structures characterized by X-ray pair distribution functional analysis.

## Introduction

Electrocatalysis is a major technical hurdle for the utilization of renewable electricity to convert CO_2_ into liquid chemical fuels. While membrane-based, vapor-fed electrolyzers can now be designed to electrolyze CO_2_ to multi-carbon products at current densities in the 1 A/cm^2^ range^1^, their efficiency is low because of high overpotentials at both the anode and cathode. At the cathode where CO_2_ is reduced, the balance of the binding energies of reaction intermediates is an important consideration for achieving high efficiency and product selectivity. Binding too weakly compromises the selectivity of CO_2_ reduction relative to hydrogen evolution, and binding too strongly poisons the electrocatalyst surface with absorbed CO. Gold, silver, and copper provide the best compromise between these extremes for single-element catalysts that reduce CO_2_ in aqueous media^2,3^. Even so, computational studies have indicated that Au, Ag, and Cu still miss the optimal balance of binding energies^4,5^. Because there are multiple reaction intermediates in the multi-electron, multi-proton CO_2_ reduction reaction (CO_2_RR), and because their adsorption energies are linearly related, tuning a catalyst surface to optimize one reaction step simultaneously affects the other steps^6^. Breaking this scaling relationship is one of the grand challenges in the development of better CO_2_RR catalysts. Numerous approaches to this problem have been proposed, including lowering the surface coordination number^7^, introducing strain and defects into the catalyst structure^8,9^, functionalizing the surface with molecules that assist in the catalytic reaction^10,11^, and controlling the electrolyte environment^12,13^.

Alloying multimetallic catalysts, in recent years, has also been demonstrated as a successful strategy for circumventing the scaling relationship. By alloying strong and weak binding elements, a catalyst with an intermediate CO binding energy can be made. For example, Kim et al. designed Au–Cu nanoparticles in similar size ranges but with different composition ratios and measured the d-band energies of the nanoparticles with high-resolution X-ray photoemission spectroscopy^14^. The changes in the AuCu catalyst performance were partially attributed to the electronic effects of the different d-band energies. In addition, incorporating elements with different affinities for carbon and oxygen can create new binding motifs for the reaction intermediates. Hansen et al. have studied the combination of transition metals and p-block elements to provide stabilization through the binding of an oxygen atom of the COOH* intermediate^15,16^. Finally, the mixing patterns of different elements can also be used to tune the selectivity of the CO_2_RR^17^. Ma et al. synthesized Cu–Pd nanoparticles in ordered, disordered, and phase-separated arrangements and observed different selectivities for each type of nanoparticle^18^. Ultimately, however, because of the complexity of the mechanistic pathways, the effects of surface composition on the selectivity of the CO_2_RR are still not completely understood.

In exploring complex catalyst composition spaces that may include both alloying elements and surface-modifying molecules, high-throughput experimentation can often accelerate the process of catalyst discovery. The utility of these methods has been demonstrated for several other problems in electrocatalysis, including methanol oxidation in direct methanol fuel cells^19,20^, the oxygen reduction reaction^21^, the oxygen evolution reaction^22–24^, the hydrogen evolution reaction (HER)^25^, and methane reforming^26^. Enabling a high-throughput method for CO_2_RR electrocatalyst discovery can be an important step toward establishing a reliable database for the study and prediction of new materials^27,28^. Theoretical studies combined a database of DFT calculations with machine learning and predicted bimetallic alloys with optimal binding sites^29^. Several reports have so far applied parallel synthetic methods to study CO_2_RR catalysts, although in general those methods have involved serial catalyst testing or testing in array potentiostats that can screen small numbers of catalysts^30–32^. One of the persistent challenges with the high-throughput screening of CO_2_RR catalysts is the suppression of the competing HER^30^. The balance between the HER and CO_2_RR is difficult to measure unless each individual catalyst tested is coupled to product analysis. Thus, large-scale and parallel screening of CO_2_RR catalysts has yet to be realized.

In this study, we adapt a high-throughput optical screening method originally developed for liquid-phase methanol electrocatalysis^19^ to study CO_2_RR electrocatalysis. Because of the high current densities that have been achieved for the CO_2_RR in gas-fed electrolyzers, we adapt this method to CO_2_ delivery via a gas diffusion electrode^1,33,34^. Alloy catalyst arrays were prepared by deposition of aqueous metal salt solutions with an automated liquid handler followed by hydrazine reduction and were screened in parallel by a fluorescence-based optical technique. We first developed a rough catalytic activity map of bimetallic CO_2_RR alloy catalysts that screened the performance trends of different chemical groups from the periodic table. A subset of active elements was then chosen to make ternary composition maps that sampled the composition space uniformly. Activity maps were made by parallel screening for the CO_2_RR and HER. Finally, for the best ternary catalysts, we evaluated the alloying of elements by X-ray diffraction and pair distribution function (PDF) analysis to understand the relationship between alloy structure and catalytic activity.

## Results

**Fig. 1:**
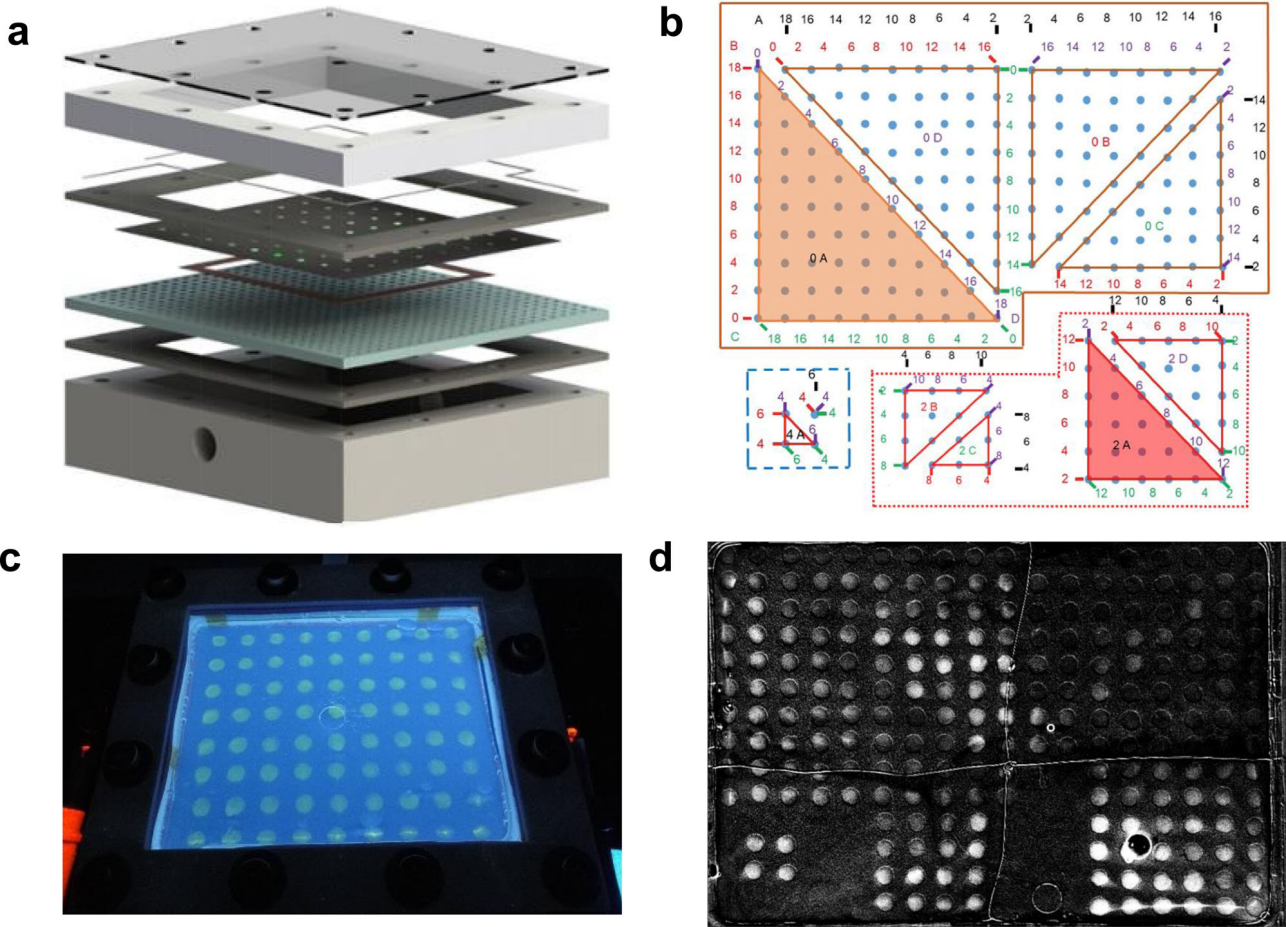
Screening cell. **a** Exploded view of the electrochemical screening cell. Pt and Ag wires are used as the counter and reference electrodes, respectively. A Cu current collector is beneath the array working electrode. The cell sandwiched between silicone gaskets and isolated from air. **b** Mapping of a four-component composition space (220 discrete compositions). **c** Color image showing the fluorescence pattern from a uniform grid of Pt catalyst spots. **d** CO_2_RR fluorescence image of a four-component electrocatalyst array.

Figure 1 shows the setup of the screening experiments. Arrays of multimetallic catalysts were robotically deposited onto Toray carbon paper to make an array working electrode, and the working electrode was then assembled into a gas-fed, three-electrode electrochemical cell that was fabricated by 3D printing (see “Methods” for cell construction, catalyst synthesis, and testing details). Different chemical compositions, as well as the geometry of the array pattern, can be accessed (Supplementary Fig. S1) by programming the deposition robot. During a screening experiment, gaseous CO_2_ flows from below the working electrode through the porous Toray carbon sheet to the catalyst spots at low positive pressure by adjusting a needle valve at the outlet (see Supplementary Videos S1 and S2). The liquid electrolyte, which covers the top side of the working electrode, is 1 M 1-ethyl-3-methylimidazolium tetrafluoroborate (EMIM^+^BF_4_^−^) and 0.5 M DI H_2_O in acetonitrile. The ionic liquids added to suppress the HER, and water is added to provide a source of protons for the CO_2_RR^35,36^. As the CO_2_RR proceeds at a catalytic surface, protons are consumed, and a locally higher pH develops in the unstirred solution immediately above the catalyst spot. A fluorescent acid-base indicator, 2,7-dichlorofluorescein, is added to the electrolyte to image the local pH increase, which provides a measure of the activity of the catalyst. The onset potential, where the fluorescence is first observed, is recorded as the descriptor of the catalytic activity.

The essence of this technique is to capture the onset potential of different alloy catalysts simultaneously in the same electrochemical environment. This enables simultaneous screening of a large number of catalysts (e.g., 220 in the four-component array shown in Fig. 1) under identical experimental conditions. Measuring the onset potential (as opposed to measuring current at higher overpotential) minimizes potential systematic errors arising from iR drop or mass transport effects. Supplementary Video S1 shows a screening experiment performed with 72 identical Pt catalysts (in an 8X9 array, Fig. 1c) in which all the catalyst spots—regardless of their distance from the reference electrode—give a similar fluorescence signal at the onset potential. This indicates that iR drop across the array does not significantly affect the measurement. In addition, the HER is a competing reaction that similarly consumes protons. Thus, all the screening experiments were repeated with N_2_ gas instead of CO_2_ to obtain background activity maps. If a catalyst is active for water reduction but not for CO_2_ reduction, or vice versa, the difference in the fluorescence onset potentials can be measured.

**Fig. 2:**
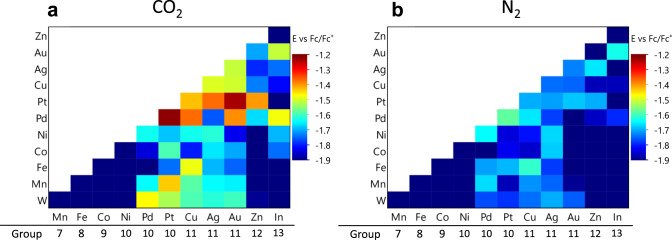
Results of survey screening. Catalytic activity map for alloyed transition metal element under (**a**) CO_2_ and (**b**) N_2_. Screening experiments were performed with 1 M ionic liquid and 0.5 M DI water in acetonitrile.

We first performed a low-resolution survey experiment to address the question of which elements might be the best candidates for alloying, and how their performance changes when they are combined with other elements. Figure 2a shows the fluorescence onset potentials of bimetallic combinations of co-reduced metals from groups 6 to 13 of the periodic table at 50:50 atomic ratio under screening with CO_2_ gas, whereas Fig. 2b shows the same experiment using N_2_ gas. In general, the onset potentials for catalysts screened under N_2_ are a few hundred mV more negative. This is consistent with earlier reports in which ionic liquids were shown to suppress the HER and to lower the activation energy for the CO_2_RR^37,38^. In Fig. 2a, binary combinations containing elements from Group 11 have more positive onset potentials than the other groups. Group 11 contains the coinage metals (Cu, Ag, Au) which are known to have high CO_2_RR activity. Thus, it is expected that alloying with Group 11 elements would produce catalysts that are active toward CO_2_RR. Other than Group 11 elements, Pt- and Pd-containing binaries were also active in the CO_2_RR survey experiments. However, both Pt and Pd are good catalysts for hydrogen evolution^39^. This is consistent with Fig. 2b, which shows that binary catalysts containing Pt or Pd are also very active for hydrogen evolution. Binary compositions containing Au, Zn, and In are also active under CO_2_ screening and show little HER activity when screened under N_2_. This observation, which is consistent with earlier reports of mixed-metal catalysts^31^, and the computational predictions of Ulissi et al.^29^, suggest that these three elements are good candidates for further study.

It is important to note that the results shown in Fig. 2 come from co-reduced binary compositions that are not necessarily homogeneous alloys; indeed, their phase behavior is likely to involve both equilibrium and nonequilibrium phases and to be particle size-dependent. We note that the 50:50 atomic ratio chosen will miss many of the catalytically interesting phases or combinations of phases among the binary combinations of these elements. Further, some of the elements surveyed (e.g., Fe, Mn, W) may not be fully reduced to metals by hydrazine. Thus, a search on a finer grid of composition space, coupled with chemical and structural analysis, is needed to develop a useful database of catalyst activity. But the capability of the optical screening method to undertake this task is clearly demonstrated with this rough survey map.

**Fig. 3:**
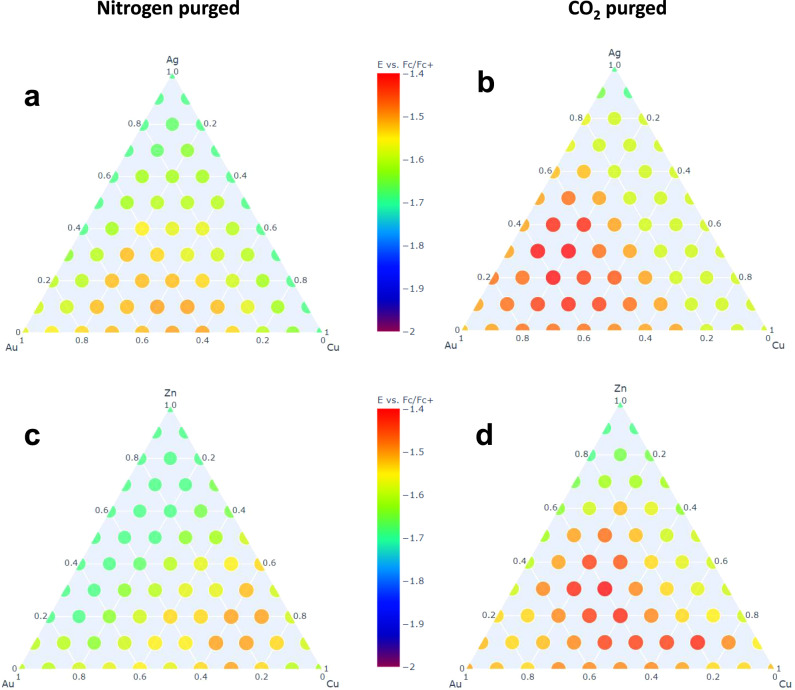
Ternary catalyst screening results. **a** In total, 66 Au–Ag–Cu compositions screened under N_2_, and **b** under CO_2_. **c** Au–Zn–Cu compositions screened under N_2_, and **d** under CO_2._ Screening experiments were performed with 1 M ionic liquid and 0.5 M DI water in acetonitrile.

In order to search ternary composition spaces for CO_2_RR catalysts, we prepared triangular arrays of discrete compositions that varied along binary lines in 10% increments, for a total of 66 different compositions. These compositions were then screened as described above. Figure 3a, b shows heatmaps, derived from the fluorescence onset potentials of discrete compositions in the Au–Ag–Cu ternary space, when screened under N_2_ and CO_2_ gas flow, respectively. The hot zone for Au–Ag–Cu ternary catalysts under CO_2_ is observed to center around Au_6_Ag_2_Cu_2_. The composition map also reveals that the Au–Cu and Au–Ag binary lines are more active than Ag–Cu. On the Au–Cu binary line, the most positive onset potential is observed around Au_7_Cu_3_, similar to a previous report of AuCu alloys^14^. Overall, the most active catalysts in the Au–Ag–Cu composition space are found in the Au-rich region. Replacing Ag with Zn in the Au–Ag–Cu ternary, we obtain the activity maps shown in Fig. 3c, d. The most positive onset potential for CO_2_ reduction is observed around Au_4_Zn_3_Cu_3_, and the onset potential is close to that of Au_6_Ag_2_Cu_2_. The most active region is observed to spread toward the Au–Cu binary line, reaffirming that Au and Cu are two of the best single-element catalysts for CO_2_RR. When we replaced Au, the last precious metal in this ternary, with In and screened Cu–Zn–In compositions, single-element Cu catalysts dominated the performance of the ternary combinations (see Supplementary Fig. S14).

The activity maps shown in Fig. 3 typically show a few hundred mV overpotential under N_2_, relative to the onset potential under CO_2_, which is consistent with the survey experiments shown in Fig. 2. This CO_2_/N_2_ comparison is important for capturing the CO_2_RR activity of the ternary compositions and eliminating false positives, in which the pH change and fluorescence result primarily from HER catalysis.

**Fig. 4:**
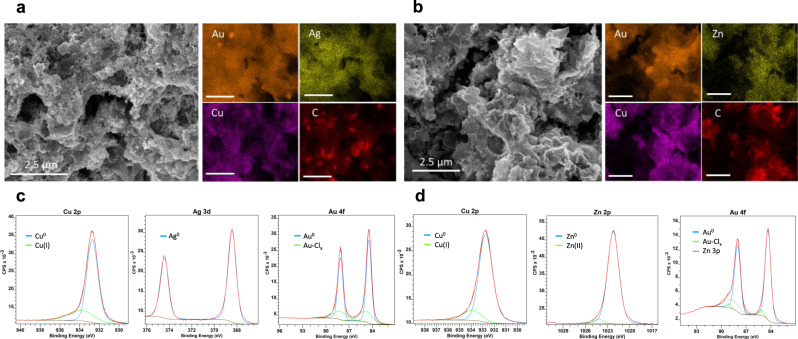
Materials characterization of the ternary catalysts. **a** SEM image and EDS spectra for Au_6_Ag_2_Cu_2_ and **b** for Au_4_Zn_3_Cu_3_. All scale bars in (**a** and **b**) are 2.5 µm. **c** Cu 2*p*, Ag 3*d*, and Au 4*f* XPS spectra for Au_6_Ag_2_Cu_2_. **d** Cu 2*p*, Zn 2*p*, and Au 4*f* XPS spectra for Au_4_Zn_3_Cu_3_.

While optical screening provides a rapid, parallel method for surveying a composition space of electrocatalysts, understanding the results requires the characterization of the materials’ chemical composition, structure, and morphology. Accordingly, we deposited the optimized ternary catalysts from Fig. 3 onto Ti foils for materials characterization. The SEM images and EDS spectra in Fig. 4a, b show the surface morphology and elemental distribution of Au_6_Ag_2_Cu_2_ and Au_4_Zn_3_Cu_3_, respectively. Both catalysts contain particles in the hundreds of nm to µm size range. EDS maps show an even elemental distribution with no apparent segregation at the sub-micron resolution of the technique. Clusters of carbon around 100 nm in diameter are likely from aggregated carbon powder or Nafion. The bulk chemical compositions were determined from the EDS analysis (Supplementary Figs. S9 and S10) as Au_0.44_Ag_0.16_Cu_0.4_ and Au_0.26_Cu_0.52_Zn_0.22_, in reasonable agreement with the intended compositions given the semi-quantitative nature of EDS. Thus, we refer to the samples hereafter by their intended deposition stoichiometry. The only other elements detected in the EDS spectra were oxygen, nitrogen, fluorine, and chlorine (Supplementary Figs. S7 and S8). Oxygen comes from metal oxide formation which we will discuss with the XPS data. Nitrogen may come from residual nitrate from the metal salt precursors or from the byproducts of hydrazine reduction. Fluorine can be assigned to Nafion entrained in the Toray paper. Chlorine likely comes from residual precursor solution retained after washing. We synthesized a bulk alloy powder for X-ray diffraction and scattering analysis in the absence of Nafion and the carbon support in order to minimize the background signal from carbon. The powder diffraction patterns (Supplementary Fig. S3) show primarily a face-centered cubic (fcc) alloy structure for both Au_6_Ag_2_Cu_2_ and Au_4_Zn_3_Cu_3_. This would suggest that Ag and Cu, or Cu and Zn, are incorporated into the fcc Au structure without forming any other crystalline phase. It should be noted however that the lattice constants of fcc Ag and Au are nearly identical, so this question is revisited in more detail with PDF analysis of diffraction data below. The fcc diffraction peaks consist of a sharp peak with broadening in the shoulder, indicating a mixture of nanoscale and larger scattering domains. The lattice constants for Au_6_Ag_2_Cu_2_ and Au_4_Zn_3_Cu_3_ were 0.4075 and 0.4065 nm, respectively, calculated from the d spacings of the XRD patterns. Both are slightly smaller than that of fcc Au (0.40782 nm) or Ag (0.40853 nm) which is likely due to the incorporation of Cu and Zn atoms into the lattice. EDS analysis of the powder samples showed that their compositions were richer in Au and contained relatively less Cu and Zn, relative to those made on carbon paper, as shown in Supplementary Figs. S9 and S10.

XPS spectra were collected on the samples deposited on carbon paper to determine the chemical states of each element on the surface of the catalysts. Figure 4c, d shows representative spectra of the metallic elements for Au_6_Ag_2_Cu_2_ and Au_4_Zn_3_Cu_3_, respectively. Both Cu and Zn show small amounts of oxide on the surface. The Cu oxide was determined to be mostly in the Cu^+^ state from the photoemission and the Auger spectra (Supplementary Fig. S11). Cu^2+^ has a strong satellite feature at around 943 eV, which is absent in the spectra. Even though the oxidation state of copper was not intentionally controlled in the synthesis, a small amount of surface Cu^+^ has been shown in several reports to be beneficial for CO_2_RR catalysis^40–42^. Ag matches the reference standard fairly well and is mostly in the zero oxidation state. A trace amount of Cl^−^ is detected on the catalyst surface for both Au_6_Ag_2_Cu_2_ and Au_4_Zn_3_Cu_3_ and is likely from residual Au precursor solution that was not washed away. Overall, the XPS data show that the elements are predominantly in their metallic state with some surface oxide formation and a residual amount of Cl^−^ on the surface.

**Fig. 5:**
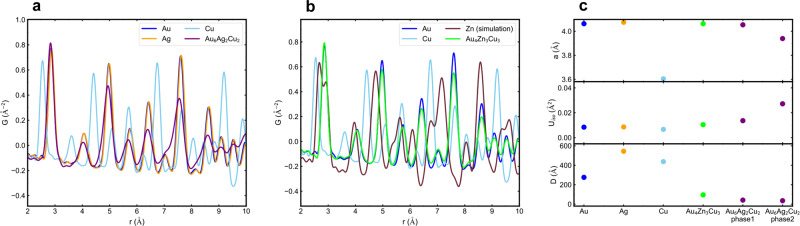
Results of atomic PDF analysis. Overlapping plot of single metal and alloy nanoparticles zoomed in to the low-r region. **a** Au_6_Ag_2_Cu_2_, **b** Au_4_Zn_3_Cu_3_. **c** Summary of fitting results. “a” is the lattice constant, “Uiso” is the isotropic ADPs, and “D” is the diameter of the crystallites. There are two phases in Au_6_Ag_2_Cu_2_, labeled as “phase1” and “phase2”.

A more detailed study of the alloy catalyst structures was performed by atomic pair distribution function analysis (PDF) and the results are shown in Fig. 5. The alloy nanoparticles were compared to nanoparticles of the individual constituent metals that were synthesized under the same conditions. The signal is not a simple linear combination of the individual metal signals, which suggests that an alloy phase is present. Faster attenuation of the PDF signal in the alloy samples compared to the pure metals indicates that the average alloy crystallite size is smaller than the pure metal nanoparticles. Three different hypotheses were modeled and compared with the measured PDF signals to see which one can represent the structure of the samples (see Supplementary Information for details). This analysis suggests that the Au_4_Zn_3_Cu_3_ are optimally characterized as a single fcc phase alloy with a lattice slightly smaller than pure Au nanoparticles but larger than Cu. Since Cu–Cu and Zn–Zn distances are both shorter than the distances between the neighboring atoms in the average structure of Au_4_Zn_3_Cu_3_, the Cu and Zn atoms leave vacancies if they are surrounded by Au atoms. This leads to disorder, which in turn causes the fitting results of atomic displacement parameters (ADP) and crystallite size to deviate from those of the pure metal nanoparticles. For the Au_6_Ag_2_Cu_2_ catalyst, a lattice similar to fcc is seen, and the best description of this was achieved by assuming that two different alloy phases make up the sample. Both phases matched closely with the fcc structure of Au, but with different lattice parameters, ADPs, and crystallite sizes. However, the conclusion that two phases are present in Au_6_Ag_2_Cu_2_ is tentative, because the extra signal may originate from the disorder in the material. However, there is no convergence of the lattice parameter and ADPs of the two Au_6_Ag_2_Cu_2_ phases when looking over a larger fitting range for r (see Supplementary Information for details), which supports the hypothesis that there are indeed two phases in the sample. The presence of unique alloy phases with lattice strain and atomic vacancies could contribute to the enhanced catalytic performance of these ternary catalysts, and follow-up studies will be needed to determine if, e.g., one of the Au_6_Ag_2_Cu_2_ phases is more active than the other, and if their compositions are different.

**Fig. 6:**
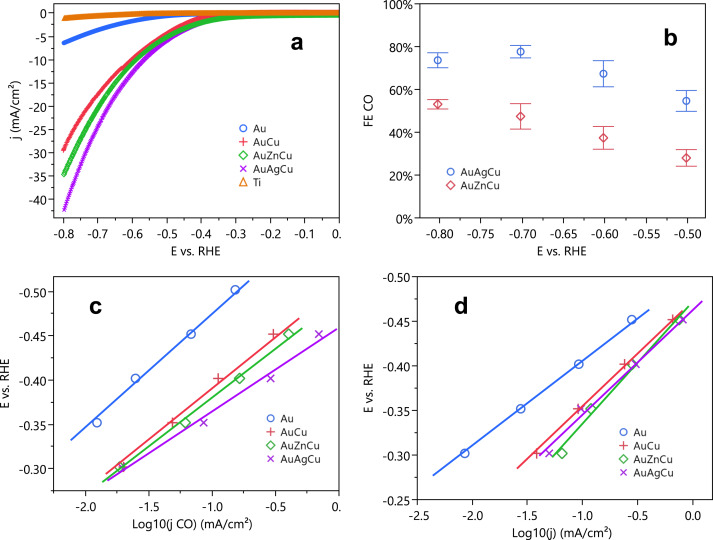
Electrochemical characterization of Au, Au_7_Cu_3_, Au_4_Zn_3_Cu_3_ and Au_6_Ag_2_Cu_2_ catalysts. **a** j–V curves; **b** CO Faradaic efficiencies; **c** Tafel plots of j–V data obtained under CO_2_ gas; and **d** N_2_ gas. All experiments were performed with a 0.5 M KHCO_3_ electrolyte. Error bars show the standard deviation of three replicate experiments, and points mark the mean value.

Electrochemical studies of the individual ternary CO_2_RR catalysts were carried out to measure product distributions and to gain mechanistic insight into the electrocatalytic reaction. Au_6_Ag_2_Cu_2_ and Au_4_Zn_3_Cu_3_ catalysts were deposited onto carbon electrodes and characterized electrochemically. The catalysts were first tested in an ionic liquid/water mixture, similar to the screening electrolyte. Supplementary Fig. S16 shows that the Au_6_Ag_2_Cu_2_ catalyst at a current density of ~30 mA/cm^2^ produced CO from CO_2_ with 90% Faradaic efficiency (FE). Au_4_Zn_3_Cu_3_ at slightly lower current density (27 mA/cm^2^) attained about 80% Faradaic efficiency. In these experiments, the current density was normalized to the geometrical area of the flat Ti substrate. A comparison of the max current density and potential onset from screening can be seen in Supplementary Fig. S17. We performed the further electrochemical analysis in KHCO_3_ solution because it is a more universal electrolyte for CO_2_RR, and because we observed stability issues with ionic liquid liquids in long term electrolysis experiments (>1 h), as noted in earlier reports^43,44^. This comparison showed a strong correlation between the screening results (measured in a nonaqueous electrolyte) with electrocatalytic activity in a bicarbonate electrolyte. Au and Au_7_Cu_3_ were added to this study to compare the effects of surface composition. The Au_7_Cu_3_ composition was chosen because it represented the best ratio on the Au–Cu binary line in their ternary screening study. All catalysts were deposited from the same total molar amounts of metal salts, and thus the number of catalytic sites for each catalyst should be roughly similar. Figure 6a shows j–V curves for these different catalysts in HCO_3_^−^ solution. The alloy catalysts showed a fourfold to fivefold increase in current density relative to elemental Au, with Au_6_Ag_2_Cu_2_ reaching up to 42 mA/cm^2^. A small decrease in CO FE was observed (Fig. 6b) in the bicarbonate electrolyte relative to the ionic liquid electrolyte, accompanied by an increase in H_2_ FE.

In order to normalize the current densities obtained with different alloy catalysts, we estimated the electrochemical surface area (ECSA) by measuring the double-layer capacitance. Representative graphs are shown in Supplementary Fig. S18, and a summary of the data is given in Supplementary Table S3. In general, Au_6_Ag_2_Cu_2_, Au_4_Zn_3_Cu_3_, and Au_7_Cu_3_ all have roughly two times higher surface area than elemental Au, which partially accounts for the higher observed current density. To gain further insight into the intrinsic activity of the catalysts, we then examined Tafel plots derived from the current-potential data. The Tafel slopes for Au, Au_7_Cu_3_, Au_4_Zn_3_Cu_3_, and Au_6_Ag_2_Cu_2_ are 128, 115, 110 mV, and 94 mV/dec, respectively (Fig. 6c). While the interpretation of Tafel slopes for CO_2_RR catalysis in bicarbonate electrolytes is complicated by local pH changes and concentration polarization effects, these effects become less problematic at low current density^45^. Polycrystalline Au has been reported to have a Tafel slope of *ca*. 120 mV/dec at high current density^46,47^, and 59 mV/dec at lower current density^45^, which is roughly consistent with our results. For comparison, Tafel plots were also made from j–V data obtained under N_2_ gas (Fig. 6d). The Tafel slopes measured for Au, Au_7_Cu_3_, Au_4_Zn_3_Cu_3_, and Au_6_Ag_2_Cu_2_ under N_2_ were 95, 118, 137, and 117, respectively. In general, while the CO_2_RR performance is improved by alloying Au–Cu with Ag or Zn, it appears that the Au_4_Zn_3_Cu_3_ and Au_6_Ag_2_Cu_2_ ternary catalysts, despite their higher surface area, are poorer HER catalysts than Au itself. This observation suggests that Zn and Ag may improve the stabilization of a CO_2_RR surface intermediate and, at the same time, inhibit the hydrogen adsorption step in HER catalysis^48^.

## Discussion

A parallel optical screening method has been successfully demonstrated for the discovery of CO_2_ reduction catalysts. The Au_6_Ag_2_Cu_2_ and Au_4_Zn_3_Cu_3_ ternary catalysts reported here are significantly more active than metallic Au catalysts or binary catalysts in the Au–Ag–Cu or Au–Zn–Cu composition spaces. The improved catalytic activity was found to originate from both the higher surface area and the intrinsic catalytic properties of the ternary catalysts. The high Faradaic efficiencies of the Au_6_Ag_2_Cu_2_ and Au_4_Zn_3_Cu_3_ ternaries derive from the fact that they are poorer catalysts for the competing HER than pure Au. A deeper understanding of why these catalysts are more effective may be discovered by confirming the structure of the catalysts on carbon paper and determining which sites are active in the CO_2_RR. It should be noted that the hydrazine reduction method used in this study results in relatively large particles and microheterogeneous distribution of elements with little chemical control. This suggests that these ternary catalysts might be further improved by exploring other synthetic techniques, such as colloidal synthesis or electrodeposition methods that favor specific crystal facets or more ideally control the catalyst morphology^40,49,50^.

The optical screening method, based on electrochemically driven pH changes, captures the onset potential as a descriptor of the catalytic reaction. Theoretical and experimental studies have shown that the onset potential is consistently correlated with catalytic activity for both CO_2_RR and HER^51–53^. While we demonstrate the screening method here as a means of exploring broad compositional ranges of alloy CO_2_RR catalysts, it should be noted that it might also be applied to evaluating catalyst modifiers, electrolyte additives, gas diffusion electrode materials, or catalyst synthetic methods with compositionally diverse electrocatalyst arrays. The method can also be used to evaluate catalyst stability over broad composition ranges, as we have previously demonstrated with five-element arrays of electrocatalysts for the oxygen evolution reaction^54^. When coupled with fast robotic synthetic techniques, the optical screening method shows promise for the generation of large datasets that could be coupled to machine learning or accelerated design of experiments.

The comparison of activity maps in CO_2_ and N_2_ rapidly differentiates catalysts that are active for the CO_2_RR and HER. However, a weakness of the optical screening method, relative to slower serial catalyst testing coupled with product analysis, is that it does not differentiate the products of CO_2_ reduction. In principle, this limitation can be overcome by utilizing fluorescent chemosensors that can detect not only local changes in pH but also changes in the local concentrations of reaction products such as CO and alcohols. Such chemosensors have already been developed for CO, formaldehyde, and other small molecules for use in biological imaging^55^ and they represent an interesting opportunity for future research in CO_2_RR electrocatalysis.

## Methods

### Electrode preparation

Catalysts were deposited onto a 5% Teflon-treated Toray 120 carbon paper (Fuel Cell Store) to make arrays of co-reduced mixed-metal catalysts. All chemicals were purchased from Sigma Aldrich and were either ACS grade or trace metal grade. All transition metal solutions were nitrate salts except Pt, Au, and W, which were H_2_PtCl_6_, HAuCl_4_, and Na_2_WO_4_, respectively. Metal salt solutions at 0.05 M were prepared with DI H_2_O/isopropyl alcohol mixture in a 3:1 volume ratio. 2 mg/ml of nano-powder carbon and 1 µL/ml of 5 wt% Nafion solution were added to the salt solutions. Carbon was added to improve the electronic conductivity, and Nafion was added as an ionomeric binder. Metal precursor solutions were prepared freshly every time as some precipitation was observed upon prolonged storage (particularly from Pt and Au solutions). The solutions were dispensed with a PipetteMax liquid handler (Gilson Inc.) onto the carbon paper in predefined ratios and combinations and were subsequently reduced with excess hydrazine. Typically, about 20 µL of the metal solutions were used to achieve a ~3-mm diameter catalyst spot on the carbon paper. The carbon paper was dried in an oven overnight at 60 °C under a nitrogen flow, washed with excess DI water, and then dried again.

The samples prepared for X-ray diffraction and atomic pair distribution function analysis were made by mixing the metal salt precursor solutions in the appropriate stoichiometry to yield the desired atomic ratio while keeping the total solution concentration at 0.05 M. The solution was stirred in a flask while excess hydrazine was added. These bulk samples were prepared in the absence of the carbon support and Nafion ionomer, and thus their compositions were not identical to those used in the optical screening and electrochemical characterization experiments. Representative EDS spectra and apparent compositions of samples prepared by the two methods are compared in Supplementary Figs. S7 and S8.

### Materials characterization

Scanning electron microscopy and energy dispersive spectroscopy were performed with a Zeiss Sigma FEI SEM. X-ray diffraction powder patterns were recorded on a PANalytical Empyrean XRD instrument with monochromatic CuKα radiation. X-ray photoelectron spectroscopy was recorded on a Physical Electronics VersaProbe II instrument with monochromatic Al Kα X-rays. The XPS spectra were calibrated with the C 1 *s* peak as 284.6 eV. All samples were prepared as previously described on a Ti foil for characterization.

### Electrochemical screening

Catalyst screening experiments were carried out in a home-made electrochemical cell (Fig. 1 and Supplementary Fig. S2). Similar to a flow type electrochemical cell, the liquid electrolyte was pooled on top of the carbon paper with the catalysts, and CO_2_ or N_2_ gas flowed under the carbon paper to create a three-phase-boundary layer during the experiment. The electrolyte consisted of 1 M 1-ethyl-3-methylimidazolium tetrafluoroborate, 0.5 M DI water, and 0.5 mM 2′,7′-dichlorofluorescein in acetonitrile. The ionic liquid was added to suppress hydrogen evolution and to also serve as the supporting electrolyte. DI water was added to provide protons for the reaction and the fluorescent pH indicator. Pt and Ag wires were used as counter and pseudo-reference electrodes and positioned in the solution a few millimeters above the carbon paper. The Ag wire was anodized in 1 M KCl solution and stored in saturated KCl when not in use. The reference potential was calibrated with ferrocene/ferrocenium (Fc/Fc^+^) solution externally prior to all experiments and was stable over time. All screening voltages were referenced to the Fc/Fc^+^ redox couple using *E*_Fc/Fc+_ = *E*_Ag wire_ − 0.486 V (see Supplementary Fig. S15 for calibration). Prior to the experiments, the electrolyte was purged with 99.99% pure CO_2_ or N_2_ gas (Praxair) for 1 h. The working electrode was pretreated at −2 mA for 10 min to reduce any oxide layer on the surface. During the experiments, CO_2_ (or N_2_) gas was flowing underneath the carbon paper at small positive pressure. The working electrode was held at constantly applied potentials between −1.1 and −1.9 V vs. Fc/Fc^+^ in 50 mV increments for 30 s each. The potential where the solution above a particular catalyst fluoresced was recorded as the onset potential. In a typical experiment, not all the catalysts induced fluorescence within the testing electrochemical window. In that case, −1.9 V was manually assigned to that catalyst to produce Figs. 2 and  3. All the screening experiments were repeated at least three times and averaged. All the electrochemical screening experiments were done with a Metrohm Autolab potentiostat (model: PGSTAT128N).

### Electrochemical characterization

Following the screening experiments, selected catalyst compositions were deposited with the same procedures onto a Ti foil for electrochemical characterization. The Ti foil was polished with 0.05-µm alumina and washed with nitric acid and then excess DI water. The electrochemical characterization was carried out in a two-compartment H-cell with Pt mesh and Ag/AgCl (saturated KCl) as the counter and reference electrodes. The two compartments were separated by a fine porous glass frit. All potentials were converted to the RHE scale according to *E*_RHE_ = *E*_Ag/AgCl_ + 0.059*pH + 0.197. Both ionic liquid (1 M EMIM^+^ and 0.5 M H_2_O in acetonitrile) and 0.5 M KHCO_3_ were tested as electrolytes. CO_2_ gas was bubbled into the electrolyte prior to the experiments for 1 h and throughout the experiment. All catalysts were first repeatedly cycled to reduce the surface oxide until their CV traces converged. Partial current densities for Tafel analysis were obtained from constant potential electrolysis experiments. The CO reduction current density was calculated from the total current density and the Faradaic efficiency. The current density during the electrolysis experiments was averaged between 5 and 10 min. Linear sweep voltammetry was collected at 20 mV/s. The double-layer capacitance was calculated from the CV traces in a non-Faradaic region of the curves at 5, 10, 25, 50, 75, and 100 mV/s. All electrochemical experiments were replicated at least three times. The CO_2_ flow rate was controlled with an MKS flow controller between 5 and 10 sccm. Product selectivity was analyzed by on-line GC sampling of the outlet gas after 10 min of reaction. The outlet gas flowed directly into a PerkinElmer 580 gas chromatography equipped with a MolSieve PLOT column and a TCD detector. The electrolyte solution after the electrolysis was also collected and analyzed on a Bruker AVIII-HD 500 MHz ^1^H NMR. No liquid products were observed. All the electrochemical characterization experiments were done with a NuVant potentiostat (model: EZStat Pro).

### PDF analysis

PDF experiments were carried out at the PDF beamline at the National Synchrotron Light Source II, NSLS-II, at Brookhaven National Laboratory, using the rapid acquisition PDF method (RAPDF^56^). A 2D PerkinElmer amorphous silicon detector was placed 204 mm behind the samples, which were loaded in 1-mm ID Kapton capillaries. The incident wavelength of the X-rays was 0.189 Å. Calibration of the experimental setup was done with Nickel as a calibrant using the program pyFAI^57^. All datasets were collected at room temperature. The detector exposure time was 60 s. Raw data were summed and corrected for polarization effects before being integrated along arcs of constant radius to produce 1D powder direction patterns using the pyFAI^57^.

Corrections were then made to the data and normalizations are carried out to obtain the total scattering structure function, F(Q), which was Fourier transformed to obtain the PDF using PDFgetX3^58^ within xPDFsuite^59^. The maximum range of data used in the Fourier transform was *Q*_max_ = 24.1 Å.

The modeling is carried out using diffpy-CMI^60^. The PDF is calculated by the histogram of atomic pair distances in structures. Three models are tested against the PDF data and the fitted PDFs are shown in Supplementary Fig. S19. Then, the PDF of Au_6_Ag_2_Cu_2_ nanoparticles is fitted by the two-phase model in a series of fit ranges. The start of the fit range is always 0 Å. The end of the fit range increases by 2.5 Å each time. The fitting results are shown in Supplementary Fig. S21.

## Supplementary information

Supplementary Information

Supplementary Movie 1

Supplementary Movie 2

Description of Additional Supplementary Files

## Data Availability

All relevant original data are available from the authors.
