# Supplementary material for: A high throughput optical method for studying compositional effects in electrocatalysts for CO2 reduction
Source: Nat Commun. 2021 Feb 18;12:1114. doi: 10.1038/s41467-021-21342-w (PMC7893049; doi:10.1038/s41467-021-21342-w)
Supplement: Supplementary file 1 — Supplementary Information [file 41467_2021_21342_MOESM1_ESM.pdf]

Supporting information for

A High Throughput Optical Method for Studying Compositional Effects in Electrocatalysts for CO_2 Reduction

Jeremy L. Hitt[A†], Yuguang C. Li[B†], Songsheng Tao[C], Zhifei Yan[A], Yue Gao[D], Simon J. L. Billinge[C,E], and Thomas E. Mallouk[A]*

[A] Department of Chemistry, The University of Pennsylvania, Philadelphia, PA 19104
[B] Department of Chemistry, University at Buffalo, The State University of New York, Buffalo, New York 14260.
[C] Department of Applied Physics and Applied Mathematics, Columbia University, New York, NY 10027.
[D] Department of Mechanical Engineering, The Pennsylvania State University, University Park, PA 16802.
[E] Condensed Matter Physics and Materials Science Department, Brookhaven National Laboratory, Upton, NY 11973.
[†] These authors contributed equally.

Video S1. Screening results with an 8X9 array of Pt catalysts. The fluorescence color was observed simultaneous for all catalysts spot indifferent to the location and distant away from the reference electrode. It demonstrates that the iR drop for the experiment is negligible due to the small current. The video is played at 5X speed.

Video S2. Sample video for the screening of Au-Ag-Cu ternary alloy under CO_2 and N_2 condition. One can observe the clear hot zone under the screening; and the different on the fluorescence onset map between CO_2 and N_2 gas. The video is played at 5X speed.

Table S2. Selected publications on multi-metallic catalysts for CO_2RR.

Catalyst	Electrolyte	j	FE	Ref.
$Au_6Ag_2Cu_2$	0.5 M $KHCO_3$	-23 mA/cm^2 @ -0.7 V vs. RHE	80% CO	This work
$Au_4Zn_3Cu_3$	0.5 M $KHCO_3$	-25 mA/cm^2 @ -0.7 V vs. RHE	50% CO	This work
AgPd nanodendrite	0.05 M $TMABF_4$	-10 mA/cm^2 @ -1.8 vs. SHE	85% CO	1
$Ag_{57}Cu_{43}$ dendrite	0.5 M $KHCO_3$	-25 mA/cm^2 @ -1.5 V vs. SCE	40% CO	2
AgCu	0.2 M KCl	-4 mA/cm^2 @ -1.2 V vs. RHE	2.5% CO<br>35% C_2H_5OH<br>10% $HCOO_-$<br>7% C_2H_4	3
Strain CuAg surface	0.05 M $CsCO_3$	-11 mA/cm^2 @ -1.05 vs. RHE	20% CO<br>10% C_2H_5OH<br>20% C_2H_4	4
AgSn core-shell	0.5 M $NaHCO_3$	-16 mA/cm^2 @ -0.8 V vs. RHE	80% $HCOO^-$<br>10% CO	5
$Au_{0.55}Pd_{0.45}$	0.1 M $KHCO_3$	-4 mA/cm^2 @ -1 V vs. RHE	5% $HCOO^-$<br>40% CO	6
AuCu ordered	0.1 M $KHCO_3$	-1.6 mA/cm^2 @ -0.77 V vs. RHE	80% CO	7
Au_3Cu	0.1 M $KHCO_3$	-3 mA/cm^2 @-0.7 V vs. RHE	65% CO<br>3% $HCOO^-$	8
CuIn nanoalloy	0.1 M $KHCO_3$	-1.5 mA/cm^2 @ -0.6 V vs. RHE	60% CO<br>10% $HCOO^-$	9
CuSn	0.1 M $KHCO_3$	-1 mA/cm^2 @ -0.6 V vs. RHE	90% CO	10
Cu/SnO_2 Core/shell	0.5 M $KHCO_3$	-10 mA/cm^2 @ -0.7 V vs. RHE	93% CO	11
CuIn	0.1M $KHCO_3$	-1 mA/cm^2 @ -0.6 V vs. RHE	90% CO	12
$Cu_{63.9}Au_{36.1}$	0.5 M $KHCO_3$	-4 mA/cm^2 @ -1.1 V vs. SCE	16% CH_3OH<br>12% C_2H_5OH	13
$Cu_{0.2}Zn_{0.4}Sn_{0.4}$	0.5 M $NaHCO_3$	-5 mA/cm^2 @ -0.6 V vs. RHE	80% CO	14
$Cu_{85}Pt_{15}$	0.5 M $KHCO_3$	-60 mA/mg @ -1.4 V vs. Ag/AgCl	30% CO	15
Ni_3Al	0.1 M K_2SO_4 @ pH 4.5	-12 mA/cm^2 @ -1.6 V vs. Ag/AgCl	35% CO<br>1% CH_3OH<br>1% $HCOO^-$<br>2% propanol	16
Ni_5Ga_3	0.1 M Na_2CO_3	-15 mA/cm^2 @ -0.6 V vs. RHE	2.5 % CH_4<br>1.5 % C_2H_6<br><1% C_2H_4	17
Pd_7Pt_3	0.1 M KH_2PO_4/ $KHPO_4$, pH 6.7	-5 mA/cm^2 @ -0.4 V vs. RHE	88% $HCOO^-$	18
Pd_7Cu_3	0.1 M $KHCO_3$	-1 mA/cm^2 @ -0.8 V vs. RHE	80% CO	19
$Sn_{56.3}Pb_{43.7}$	0.5 M $KHCO_3$	-45 mA/cm^2 @ -2 V vs. Ag/AgCl	80% $HCOO^-$	20
MoS_2 edges	$EMIM^+$:H_2O 96:4 mol%	-65 mA/cm^2 @ -0.764 V vs. RHE	98% CO	21

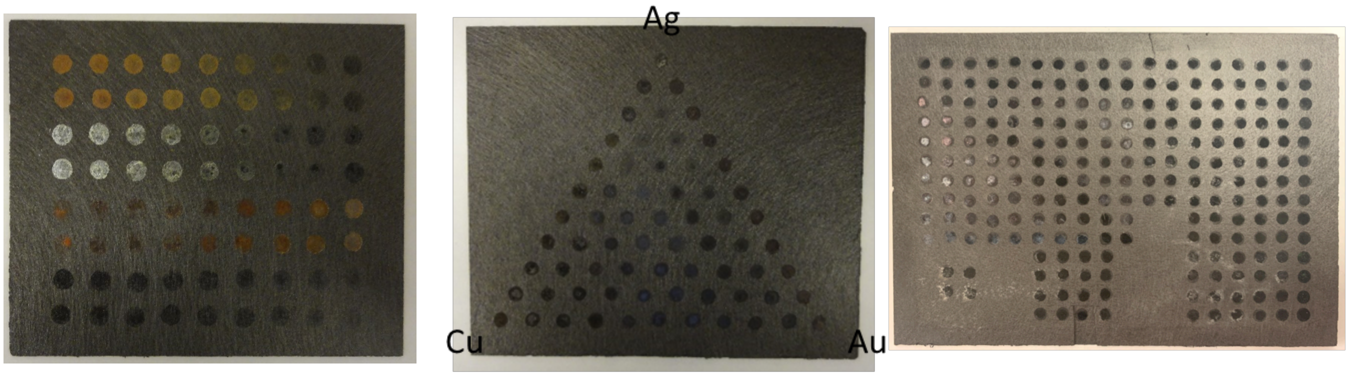

Fig. S1. Sample catalyst array with a) spots of different chemical composition, b) the ternary mapping geometry and c) the quaternary mapping geometry. Both the chemical compositions and the geometry of the array pattern can be re-programmed using the robotic plotter.

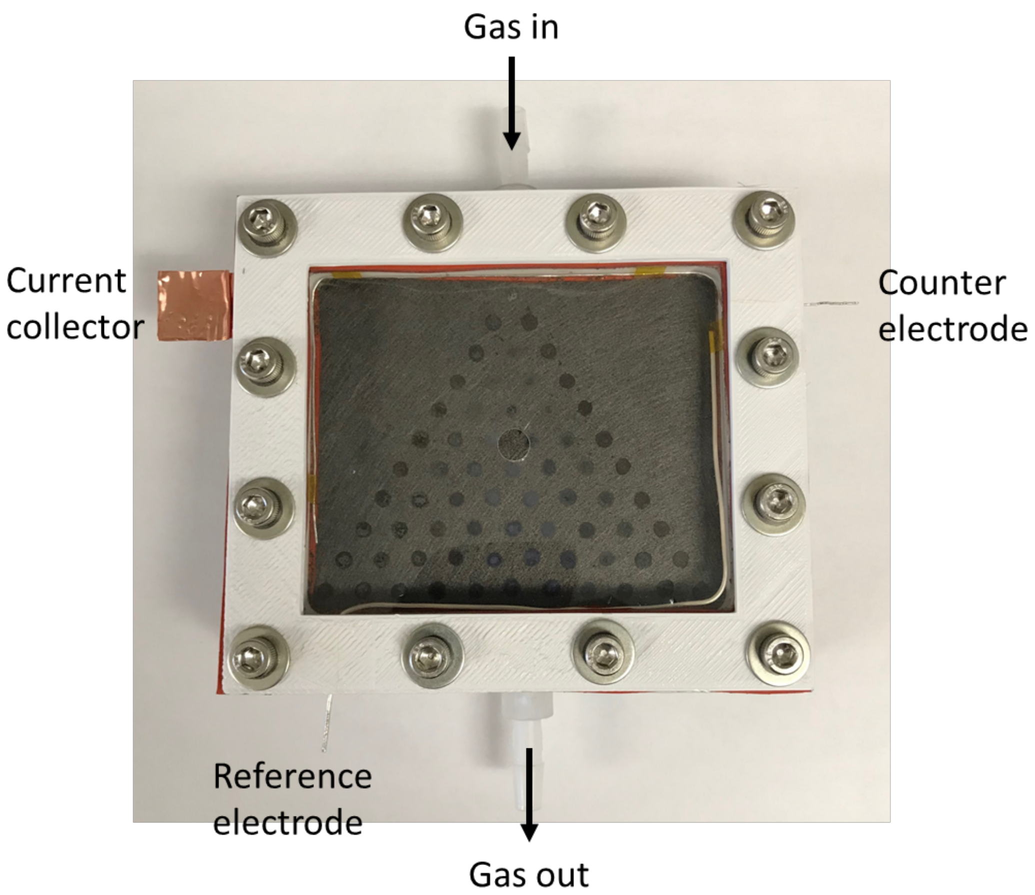

Fig. S2. Top view of an electrochemical cell for a ternary screening experiment. Counter and reference electrodes were Pt and Ag wires respectively. Copper foil was the current collector and wasinserted underneath the working electrode. The copper foil does not come into contact with the electrolyte during the screening experiment. Gas (CO_2 or N_2) flows in and out of the electrochemical cell through two fittings beneath the working electrode. The gas pressure was adjusted with a needle valve downstream to create a small positive pressure and form the three-phase-boundary layer. The housing material that comes into contact with the electrolyte is made from Teflon. The different layers are sealed with silicone gaskets and the cell is covered with a polyester film on top to isolate the working compartment from air. The hole in the polyester film is for electrolyte filling and it is sealed with transparent tape during screening experiments.

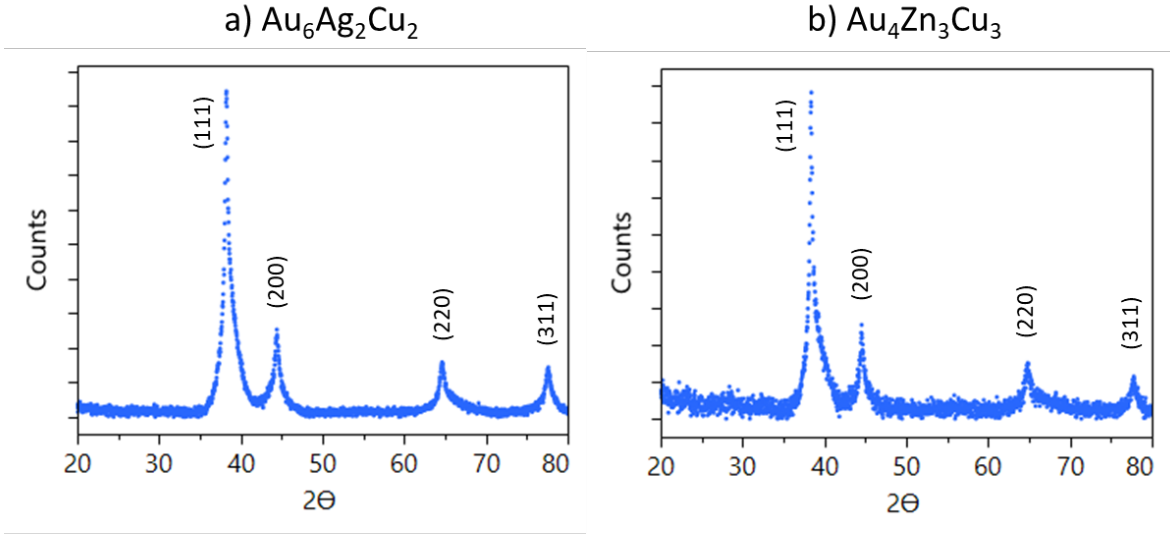

Fig. S3. X-ray diffraction patterns of a) $Au_6Ag_2Cu_2$ and b) $Au_4Zn_3Cu_3$ made with the large scale synthesis.

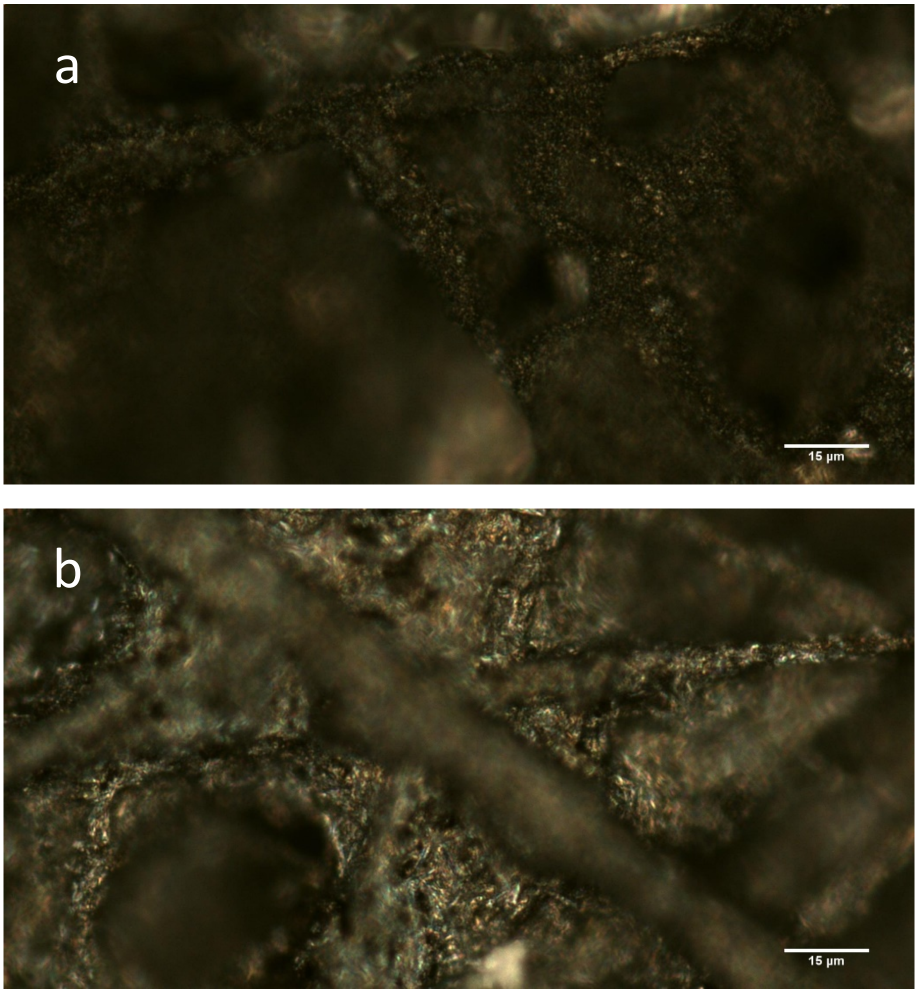

Fig. S4. Optical images of a) $Au_6Ag_2Cu_2$ and b) $Au_4Zn_3Cu_3$ on the carbon paper.

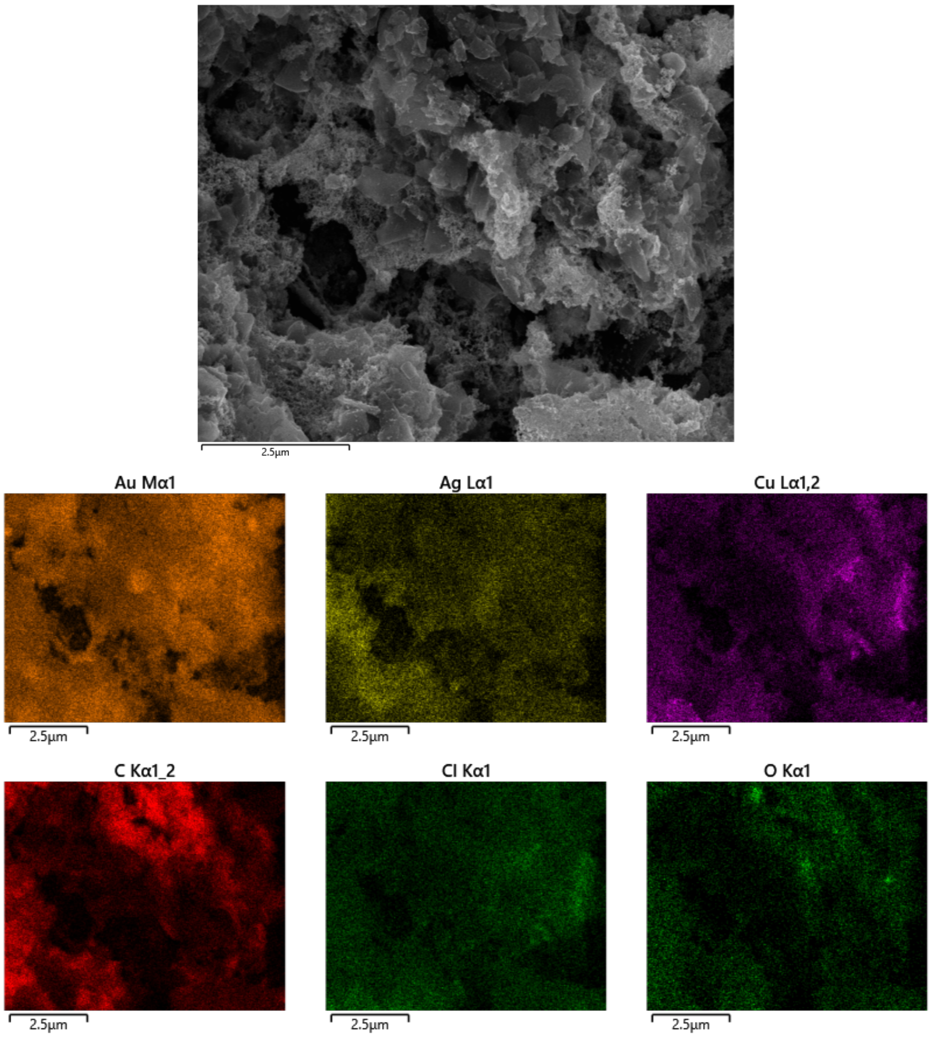

Fig. S5. Additional SEM images and EDS spectral maps of $Au_6Ag_2Cu_2$ with O and Cl signals.

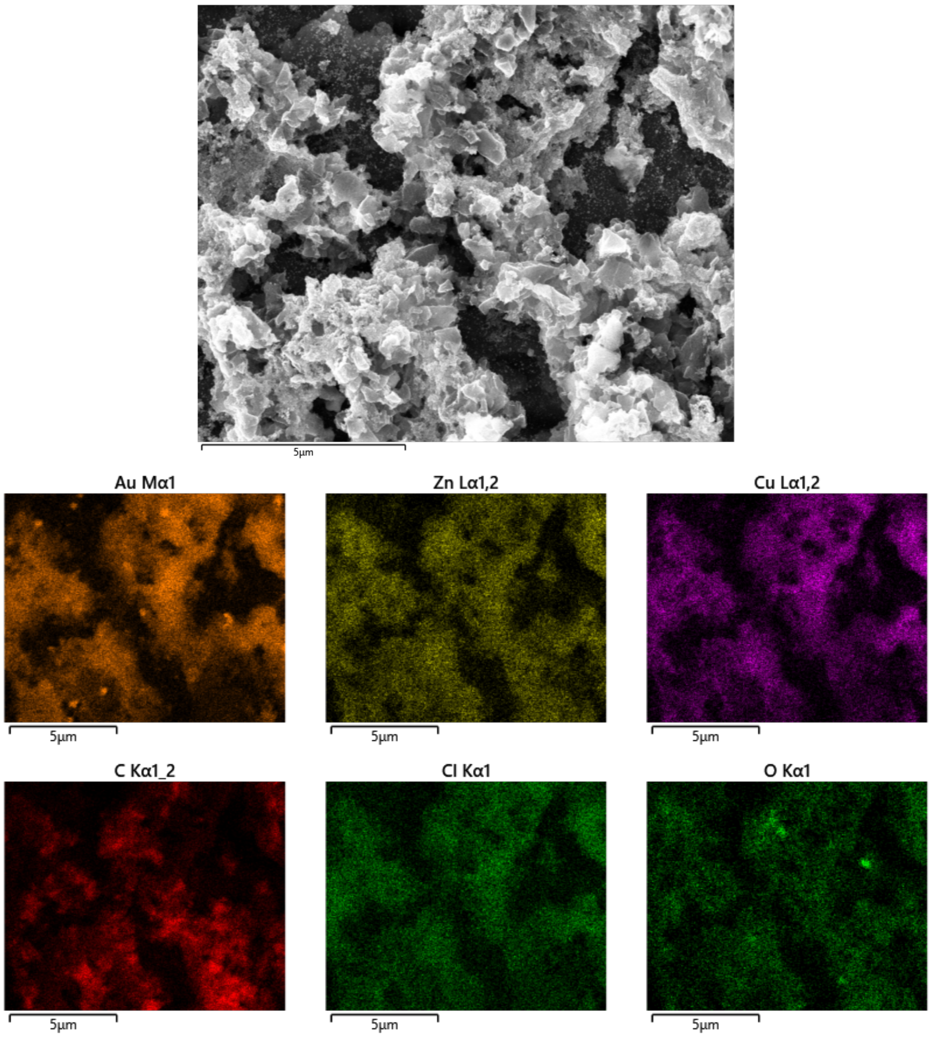

Fig. S6. Additional SEM images and EDS spectral maps of $Au_4Zn_3Cu_3$ with O and Cl signals.

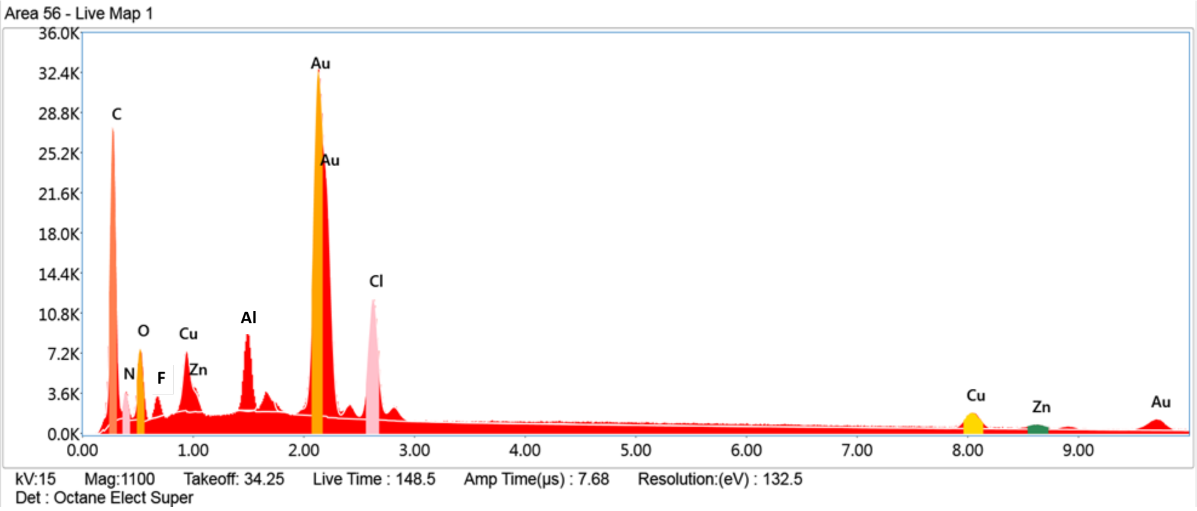

Fig S7. EDS spectrum of $Au_4Cu_3Zn_3$ alloy synthesized on Toray carbon paper by the procedure used in high-throughput screening. This spectrum gave an apparent composition of $Au_{0.43}Cu_{0.41}Zn_{0.16}$. Al, F, and some of the C signal come from the sample holder and carbon paper.

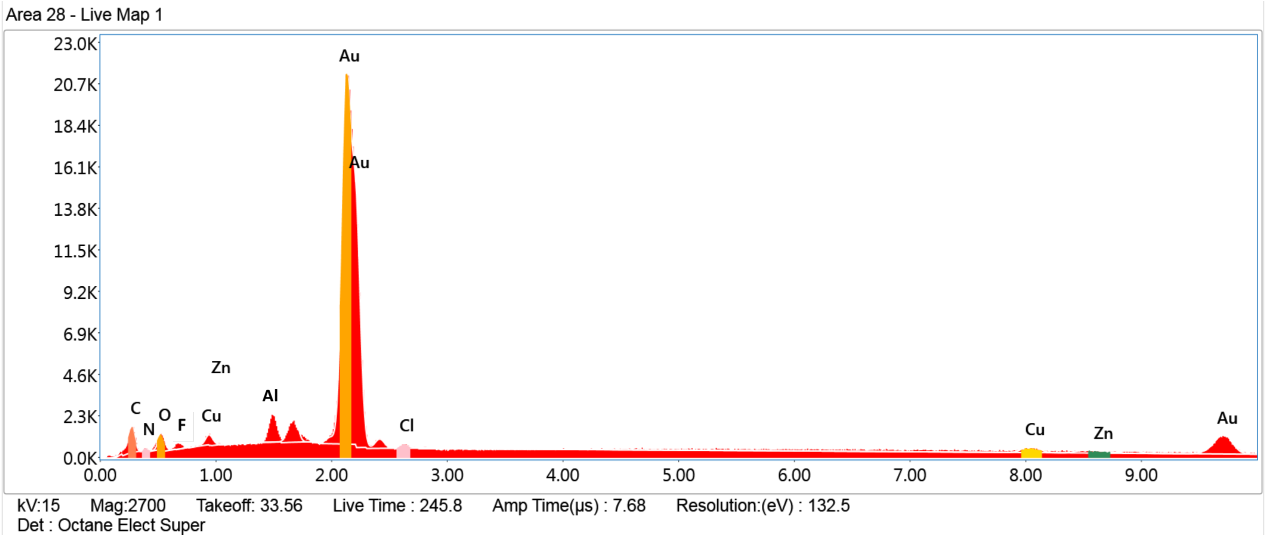

Fig S8. EDS spectrum of the $Au_4Cu_3Zn_3$ target composition, synthesized by the bulk preparation method without carbon or Nafion and with a large amount of solvent. This spectrum gave an apparent composition of $Au_{0.73}Cu_{0.20}Zn_{0.07}$. Al and some of the C signal come from the sample holder.

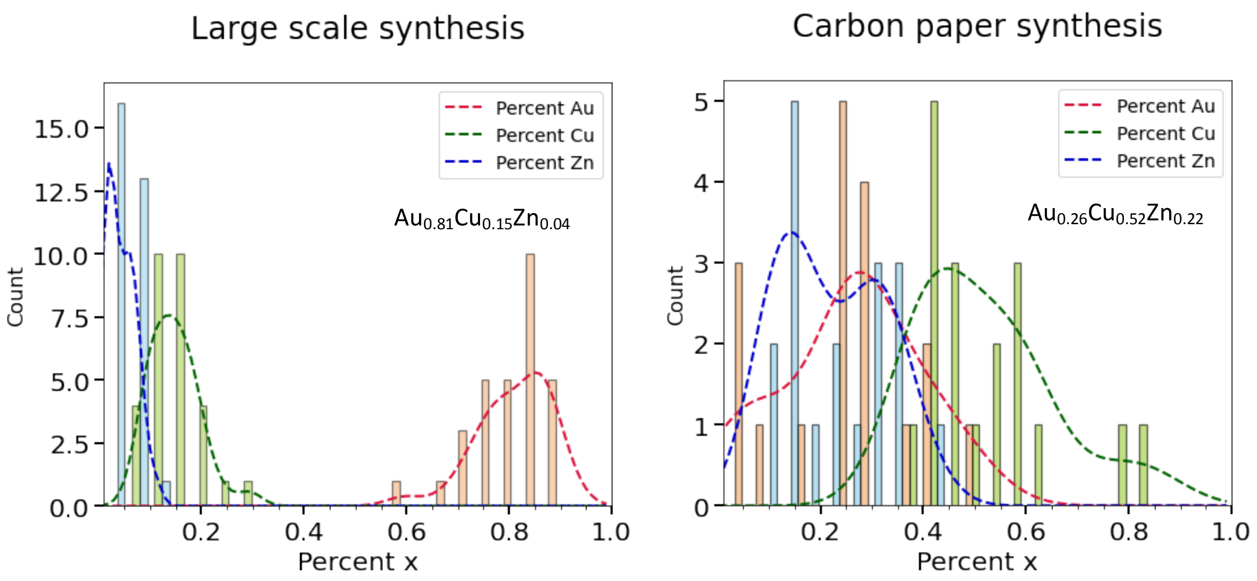

Fig S9. Distributions of the composition percentage from EDS of each element in the $Au_4Cu_3Zn_3$ alloy with fitted Gaussian kernel density estimates. a) Large scale synthesis without carbon or Nafion was synthesized in a much more dilute concentration than b) which was made by the method used in high-throughput, catalyst screening. The average composition in a) is $Au_{0.81}Cu_{0.15}Zn_{0.04}$ and the average composition in b) is $Au_{0.26}Cu_{0.52}Zn_{0.22}$.

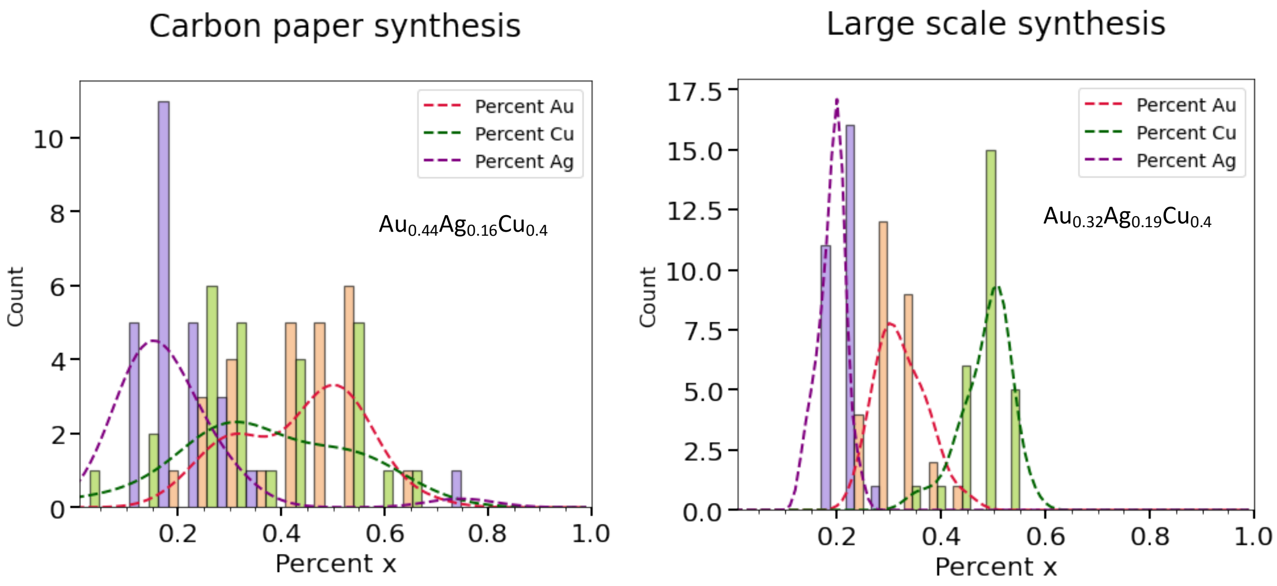

Fig S10. Distributions of the composition percentage from EDS of each element with fitted Gaussian kernel density estimates for the $Au_6Ag_2Cu_2$ alloy. The average composition in a) is $Au_{0.44}Ag_{0.16}Cu_{0.4}$ and the average composition in b) is $Au_{0.32}Ag_{0.19}Cu_{0.49}$.

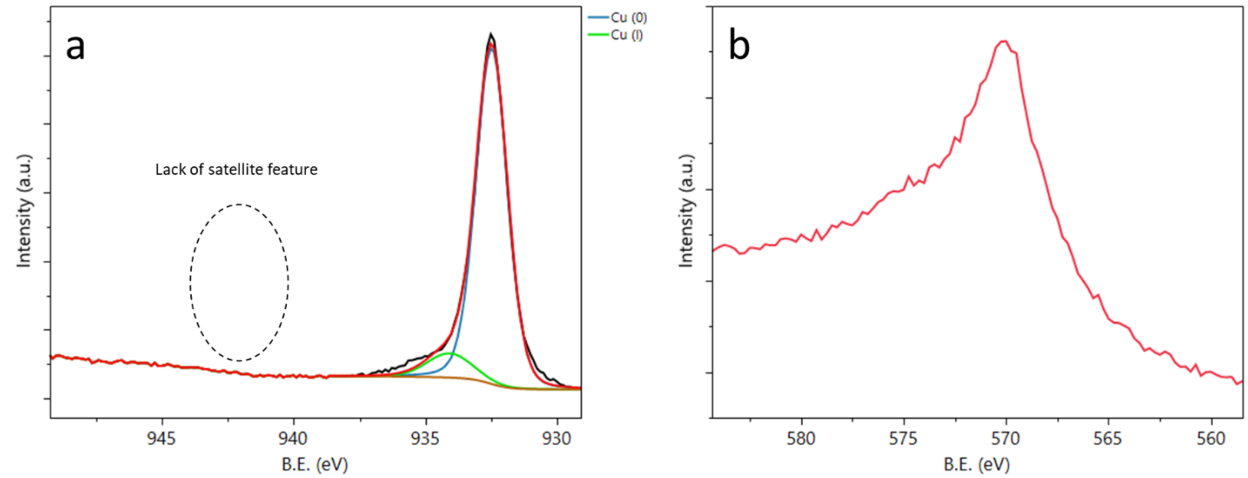

Fig. S11. XPS spectra in the Cu regions of a $Au_6Ag_2Cu_2$ catalyst. a) Expanded view of the Cu *2p* region. b) Cu LMM region. The pure Cu LMM binding energy should be 568 eV, with narrower full width at half-maximum (FWHM). The Cu LMM maximum is shifted by ~2 eV and the FWHM is broad, and thus, a surface oxide is present. Cu^{2+} photoemission spectra should have a strong satellite feature at around 943 eV, which is absent in (a). Thus, surface Cu is mostly present as the metal with a small amount of Cu^{+}. Similar spectra were observed for the $Au_4Zn_3Cu_3$ catalyst.

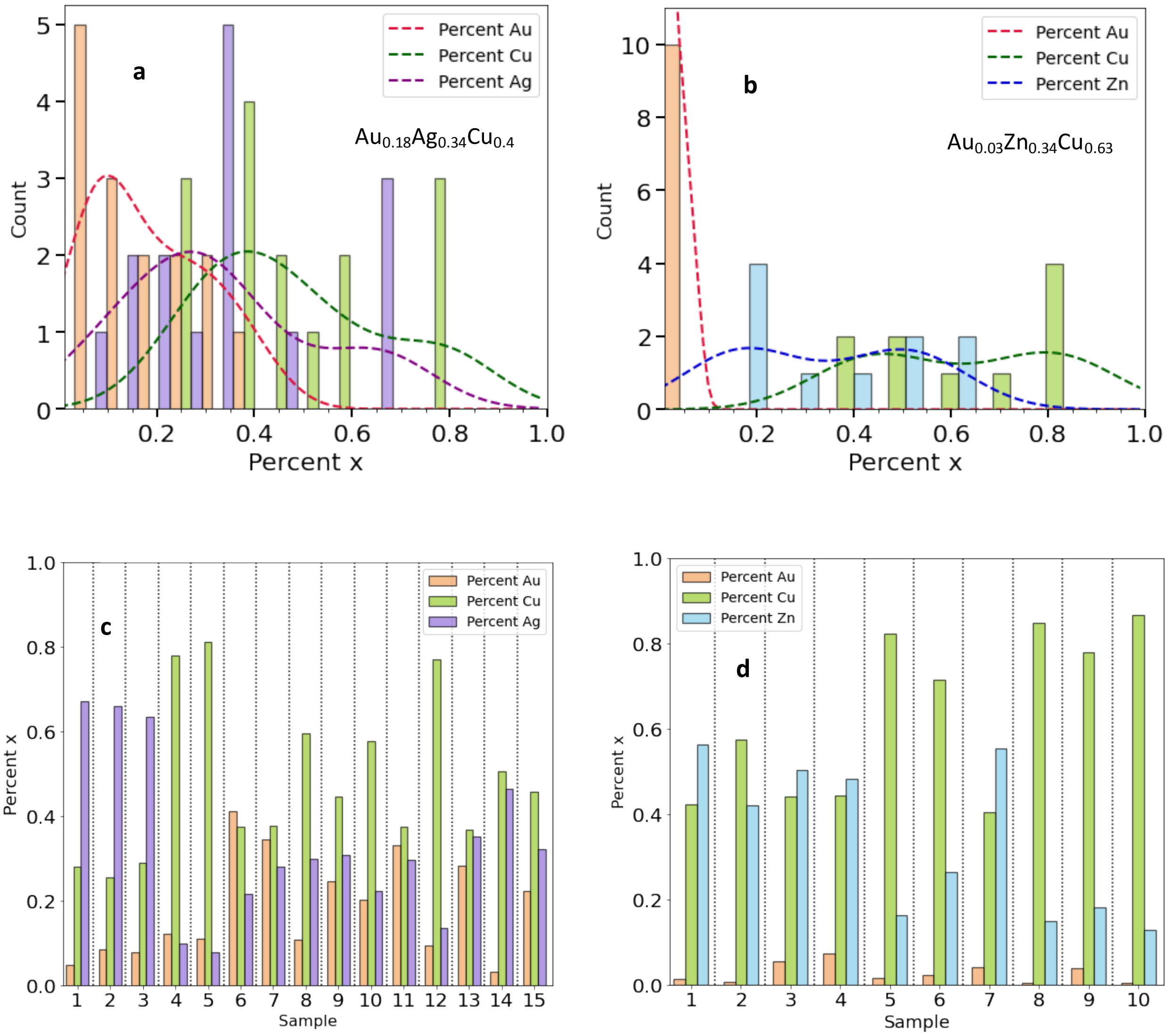

Fig. S12. Histograms with fitted Gaussian kernel density estimates of elemental composition for a) $Au_6Ag_2Cu_2$ and b) $Au_4Zn_3Cu_3$ alloys measured by XPS. The composition of each measured spot is shown in c and d. The average composition of the ***surface*** measured by XPS is $Au_{0.18}Ag_{0.34}Cu_{0.48}$ and $Au_{0.03}Zn_{0.34}Cu_{0.63}$.

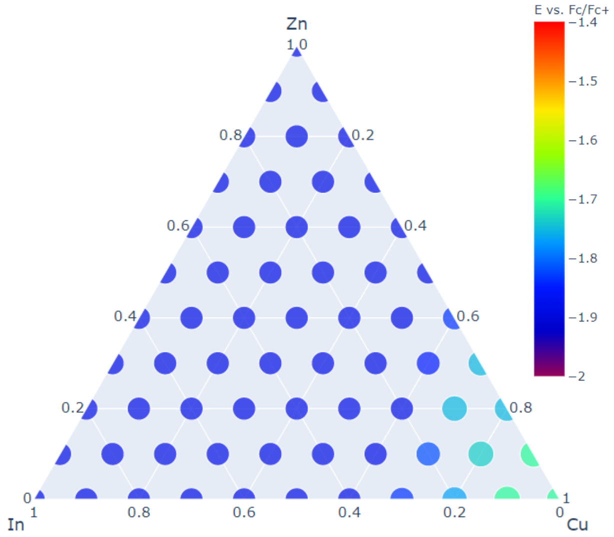

Fig. S13. Screening results for Cu-Zn-In ternary catalysts under CO_2. The Cu vertex of the activity map dominates the catalytic performance of this ternary.

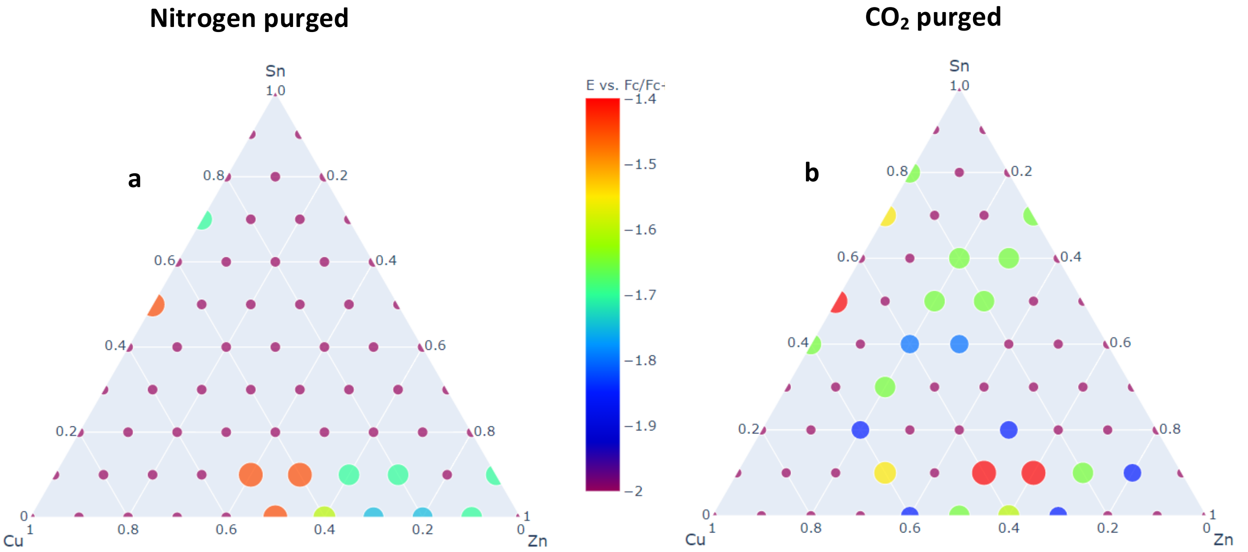

Fig. S14. Screening results for Cu-Sn-Zn ternary catalysts under N_2 (left) and CO_2 (right). Ternary compositions that are predominantly Cu and Zn have the lowest onset overpotential, but their onset overpotentials are higher than those of Au-containing ternaries.

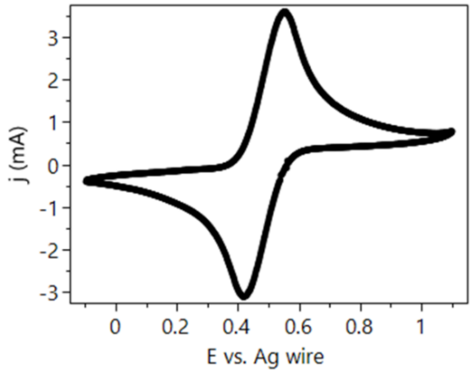

Fig. S15. Calibration CV of the Ag wire pseudo-reference electrode. Ag wire was anodized in 1 M KCl solution and stored in saturated KCl when not in used. During calibration, Ag wire was used as the reference electrode and two Pt mesh electrodes was used as the counter and working electrodes. The same electrolyte was used as the screening experiment with the addition of the Fc/Fc^{+} redox couple. Ag wire was calibrated before each experiment and was consistent over the duration of the measurements.

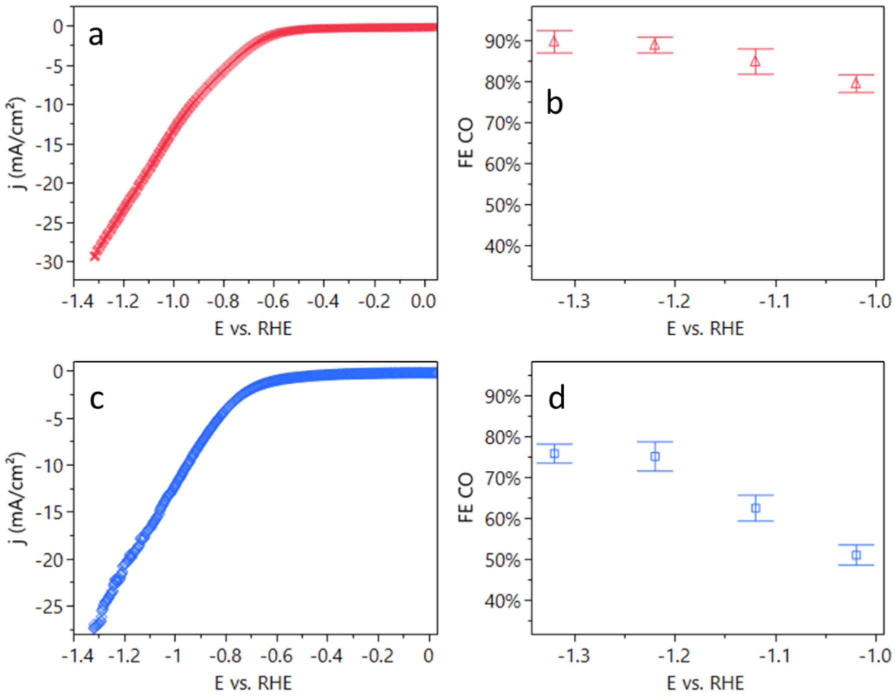

Fig. S16. Electrochemical performance of the ternary catalysts in the screening electrolyte. Current density for a) $Au_6Ag_2Cu_2$ and c) $Au_4Zn_3Cu_3$. Faradaic efficiency for b) $Au_6Ag_2Cu_2$ and d) $Au_4Zn_3Cu_3$. All data were collected in ionic liquid electrolytes (1 M $EMIM^+$ and 0.5 M DI H_2O mixture in acetonitrile). Linear sweep voltammetry was collected at 20 mV/s and Faradaic efficiency was calculated from the potentiostatic experiments by analyzing the gas output. Error bars show the standard deviation of three replicate experiments and points mark the mean value.

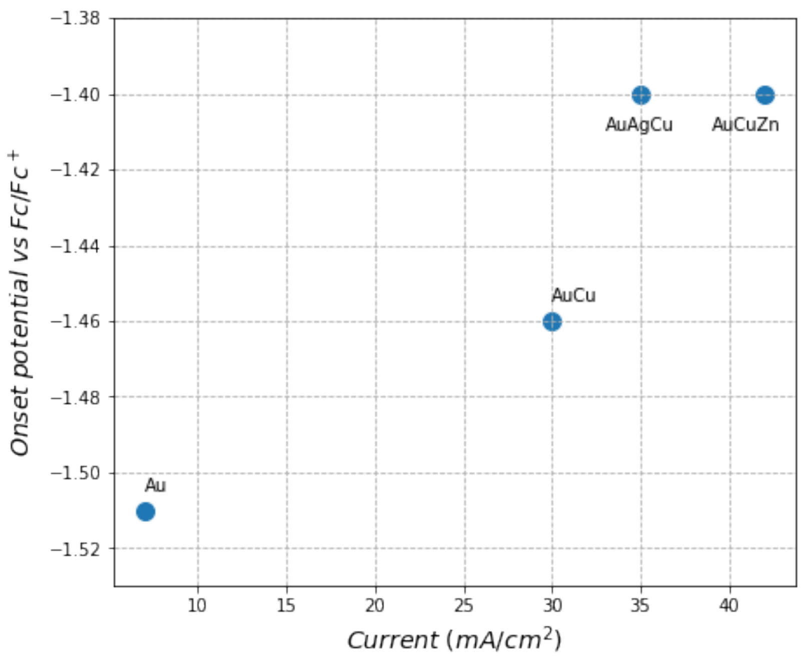

Fig. S17. A plot of the onset potential from the screening experiment (Fig 3) vs. the current density at -0.8 V vs. RHE, measured in the H-cell experiments (Fig 6) shows the correlation between the screening and testing results

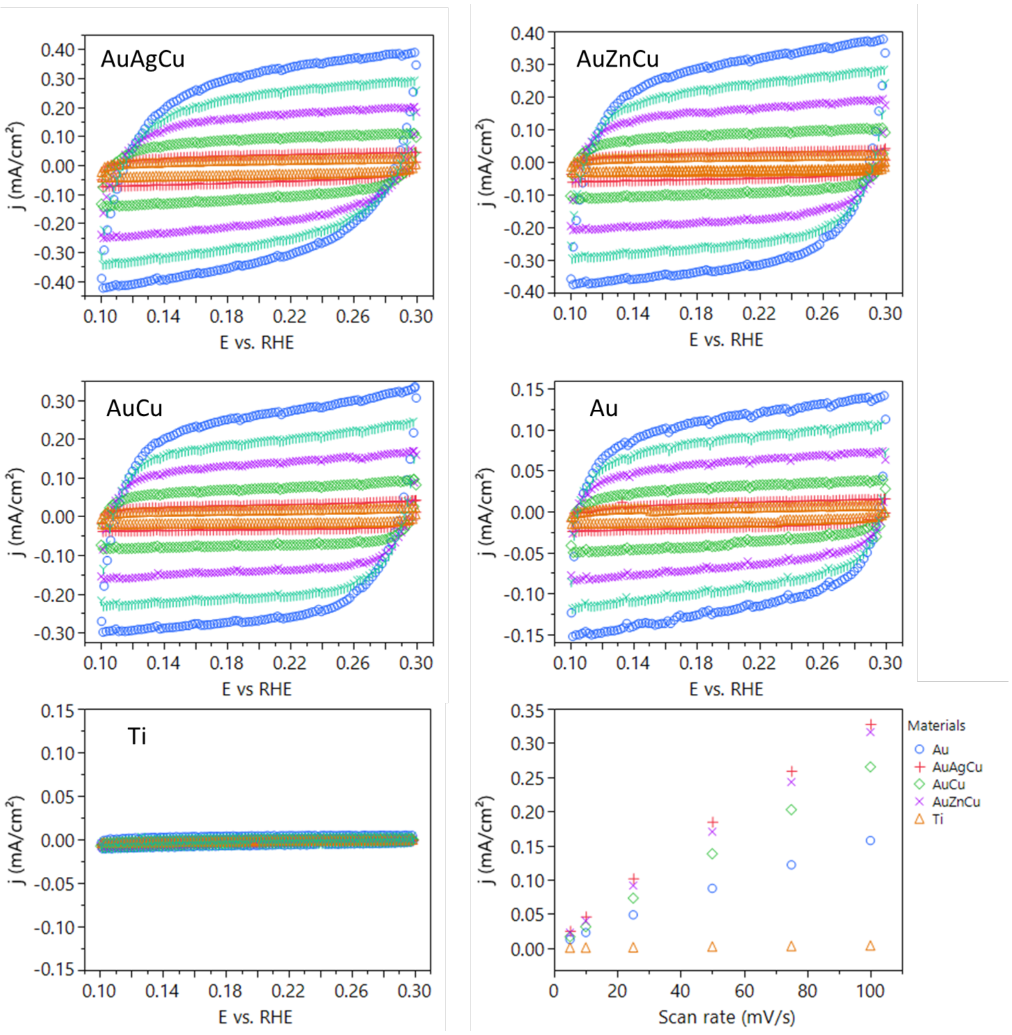

Fig. S18. Representative CV traces at different scan rates of the alloyed catalysts for calculating the double layer capacitance. Scan rates were 5, 10, 25, 50, 75 and 100 mV/s for all the materials. The last graph shows a linear fit between the scan rate and the current density. All data were collected in 0.5 M $KHCO_3$ solution. The averaged values are shown in Table S3.

**Materials**	**Capacitance (mF)**	**Std (mF)**
Au	1.4	0.2
Au_7Cu_3	2.4	0.8
$Au_6Ag_2Cu_2$	3.3	0.5
$Au_4Zn_3Cu_3$	2.5	0.9

Table S3. Double layer capacitance values of the different alloyed catalysts.

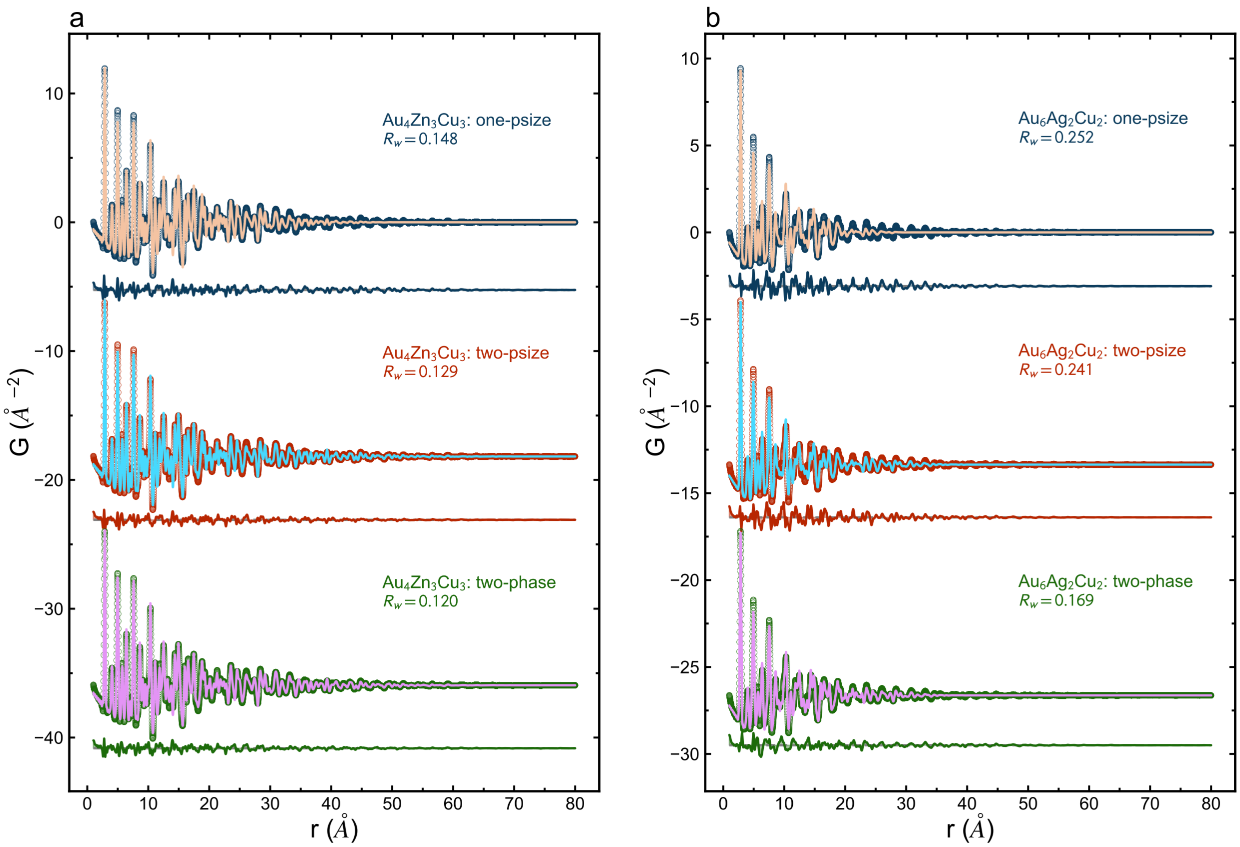

Fig. S19. The comparison between the fits of three models for PDFs of the sample (a) $Au_4Zn_3Cu_3$ (b) $Au_6Ag_2Cu_2$. The "R_w" is the goodness of the fit. The name of each model is annotated at the right of the curves. The "one-psize" model is the fcc alloy crystal PDF attenuated by a spherical characteristic function. The "two-psize" model is the fcc alloy crystal PDF attenuated by a linear combination of two spherical characteristic functions. The "two-phase" model is the linear combination of two fcc alloy crystal phases PDFs, each attenuated by a spherical characteristic function. The optimal model for $Au_4Zn_3Cu_3$ is the "two-psize" model because it has much better fits than "one-psize" model while the "two-phase" model yields negligible improvement in fits and similar lattice parameters in the two phases. The optimal model for the $Au_6Ag_2Cu_2$ is the "two-phase" model because it yields significant improvement in the fits and distinct lattice parameters in the two phases.

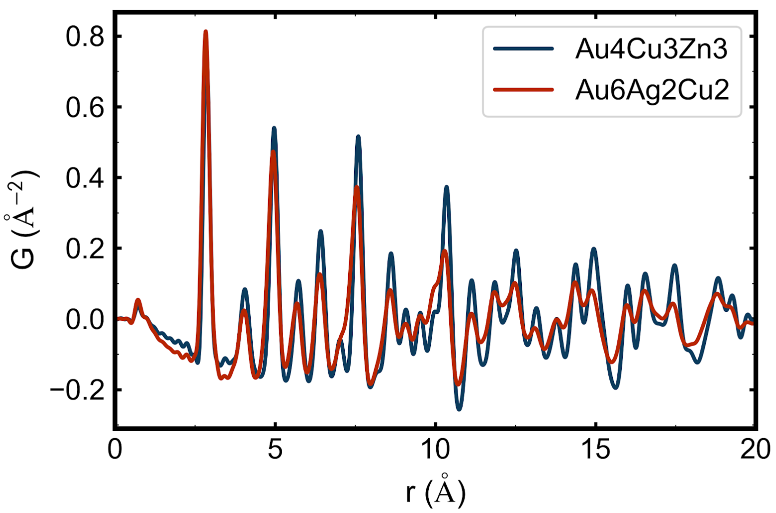

Fig. S20. Overlay of the PDF signal from the two alloys. We can see that there is more disorder (broader peaks) in the $Au_6Ag_2Cu_2$ than the $Au_4Cu_3Zn_3$.

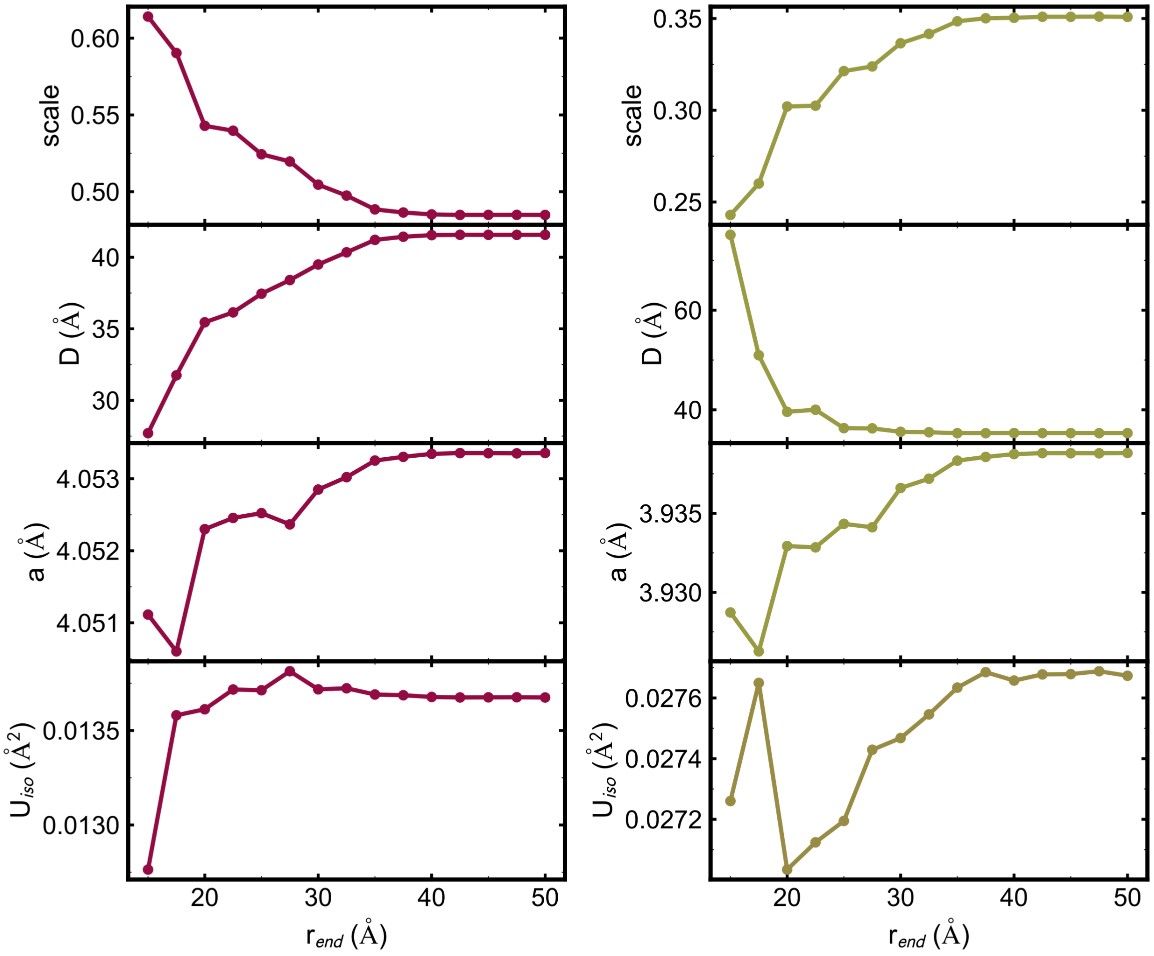

Fig. S21. Fitting results of the PDF of $Au_6Ag_2Cu_2$ nanoparticles as a function of the end point of the fitting range. The left and right panel shows the structure parameters refined in the fitting for two phases in $Au_6Ag_2Cu_2$ respectively. The "scale" is the scale factor of the PDF, the "D" is the diameter of the spherical shape crystallites, the "a" is the lattice constant, the "U_{iso}" is the isotropic displacement parameter.

References

1. Shan, C., Martin, E. T., Peters, D. G. & Zaleski, J. M. Site-selective growth of AgPd nanodendrite-modified Au nanoprisms: High electrocatalytic performance for CO_2 reduction. *Chem. Mater*. **29**, 6030-6043 (2017).
2. Choi, J. et al. Electrochemical CO_2 reduction to CO on dendritic Ag-Cu electrocatalysts prepared by electrodeposition. *Chem. Eng. J.* **299**, 37-44 (2016).
3. Lee, S., Park, G. & Lee, J. Importance of Ag–Cu biphasic boundaries for selective electrochemical reduction of CO_2 to ethanol. *ACS Catal*. **7**, 8594-8604 (2017).
4. Clark, E. L., Hahn, C., Jaramillo, T. F. & Bell, A. T. Electrochemical CO_2 reduction over compressively strained CuAg surface alloys with enhanced multi-carbon oxygenate selectivity. *J. Am. Chem. Soc*. **139**, 15848-15857 (2017).

5. Luc, W. et al. Ag–Sn bimetallic catalyst with a core–shell structure for CO_2 reduction. *J. Am. Chem. Soc*. **139**, 1885-1893 (2017).
6. Hahn, C. et al. Synthesis of thin film AuPd alloys and their investigation for electrocatalytic CO_2 reduction. *J. Mater. Chem. A* **3**, 20185-20194 (2015).
7. Kim, D. et al. Electrochemical activation of CO_2 through atomic ordering transformations of AuCu nanoparticles. *J. Am. Chem. Soc*. **139**, 8329-8336 (2017).
8. Kim, D., Resasco, J., Yu, Y., Asiri, A. M. & Yang, P. Synergistic geometric and electronic effects for electrochemical reduction of carbon dioxide using gold–copper bimetallic nanoparticles. *Nat. Comm*. **5**, (2014).
9. Larrazábal, G. O., Martín, A. J., Mitchell, S., Hauert, R. & Pérez-Ramírez, J. Enhanced reduction of CO_2 to CO over Cu–In electrocatalysts: Catalyst evolution is the key. *ACS Catal*. **6**, 6265-6274 (2016).
10. Sarfraz, S., Garcia-Esparza, A. T., Jedidi, A., Cavallo, L. & Takanabe, K. Cu–Sn bimetallic catalyst for selective aqueous electroreduction of CO_2 to CO. *ACS Catal*. **6**, 2842-2851 (2016).
11. Li, Q. et al. Tuning Sn-catalysis for electrochemical reduction of CO_2 to co via the core/shell Cu/SnO_2 structure. *J. Am. Chem. Soc*. **139**, 4290-4293 (2017).
12. Rasul, S. et al. A highly selective copper-indium bimetallic electrocatalyst for the electrochemical reduction of aqueous CO_2 to CO. *Angew. Chem. Int. Edit*. **54**, 2146-2150 (2015).
13. Jia, F., Yu, X. & Zhang, L. Enhanced selectivity for the electrochemical reduction of CO_2 to alcohols in aqueous solution with nanostructured Cu–Au alloy as catalyst. *J. Power Sources* **252**, 85-89 (2014).
14. He, J., Dettelbach, K. E., Huang, A. & Berlinguette, C. P. Brass and bronze as effective CO_2 reduction electrocatalysts. *Angew. Chem. Int. Ed*. **56**, 16579-16582 (2017).
15. Zhao, X., Luo, B., Long, R., Wang, C. & Xiong, Y. Composition-dependent activity of Cu-Pt alloy nanocubes for electrocatalytic CO_2 reduction. *J. Mater. Chem. A* **3**, 4134-4138 (2015).
16. Paris, A. R. & Bocarsly, A. B. Ni–Al films on glassy carbon electrodes generate an array of oxygenated organics from CO_2. *ACS Catal*. 7, 6815-6820 (2017).
17. Torelli, D. A. et al. Nickel–gallium-catalyzed electrochemical reduction of CO_2 to highly reduced products at low overpotentials. *ACS Catal*. **6**, 2100-2104 (2016).
18. Kortlever, R., Peters, I., Koper, S. & Koper, M. T. M. Electrochemical CO_2 reduction to formic acid at low overpotential and with high faradaic efficiency on carbon-supported bimetallic Pd–Pt nanoparticles. *ACS Catal*. **5**, 3916-3923 (2015).
19. Li, M. et al. Mesoporous palladium-copper bimetallic electrodes for selective electrocatalytic reduction of aqueous CO_2 to CO. J. Mater. Chem. A **4**, 4776-4782 (2016).
20. Choi, S. Y., Jeong, S. K., Kim, H. J., Baek, I. H. & Park, K. T. Electrochemical reduction of carbon dioxide to formate on tin-lead alloys. *ACS Sustain. Chem. Eng*. **4**, 1311-1318 (2016).
21. Asadi, M. et al. Robust carbon dioxide reduction on molybdenum disulphide edges. *Nat. Commun.* **5**, 4470 (2014).
