# Supplementary material for: A high throughput optical method for studying compositional effects in electrocatalysts for CO2 reduction
Source: Nat Commun. 2021 Feb 18;12:1114. doi: 10.1038/s41467-021-21342-w (PMC7893049; doi:10.1038/s41467-021-21342-w)
Supplement: Supplementary file 4 — Description of Additional Supplementary Files [file 41467_2021_21342_MOESM4_ESM.docx]

Description of Additional Supplementary information

Title: Supplementary Video 1

Description: Screening results with an 8X9 array of Pt catalysts. The fluorescence color was observed simultaneous for all catalysts spot indifferent to the location and distant away from the reference electrode. It demonstrates that the iR drop for the experiment is negligible due to the small current. The video is played at 5X speed.

Title: Supplementary Video 2

Description: Sample video for the screening of Au-Ag-Cu ternary alloy under CO2 and N2 condition. One can observe the clear hot zone under the screening; and the different on the fluorescence onset map between CO2 and N2 gas. The video is played at 5X speed.
